# Mapping the global prevalence and socioecological drivers of child sexual abuse: a systematic review and synthesis

**DOI:** 10.1136/bmjpo-2025-004423

**Published:** 2026-04-03

**Authors:** Bipin Adhikari, Aaryan Dahal, Abhijit Mishra, Salum Mshamu, Nipun Shrestha, Lorenz von Seidlein, Frédérique Vallières

**Affiliations:** 1 Mahidol-Oxford Tropical Medicine Research Unit, Faculty of Tropical Medicine, Mahidol University, Bangkok, Thailand; 2 Centre for Tropical Medicine and Global Health, Nuffield Department of Medicine, University of Oxford, Oxford, UK; 3 CSK Research Solutions, Mtwara, Tanzania; 4 Academy for Data Sciences and Global Health, Kathmandu, Nepal; 5 Trinity Centre for Global Health, Trinity College Dublin, Dublin, Ireland

**Keywords:** Child Abuse, Child Health, Health services research, Low and Middle Income Countries, Qualitative research

## Abstract

**Introduction:**

Child sexual abuse (CSA) is a widespread global health concern with lasting impacts. CSA is multifactorial and layered, and interventions to tackle CSA require an analysis of the key contributing factors. The main objective of this study was to explore global prevalence estimates of CSA and key contributing factors.

**Methods:**

We systematically searched PubMed, Scopus and Web of Science for studies reporting lifetime prevalence of contact (eg, inappropriate touching or sexual acts) and non-contact (eg, exposure to sexual content or ill-intended conversations) CSA. Of 5493 articles screened, 332 met inclusion criteria. Prevalence data were stratified by population, region and abuse type. Risk factors were synthesised thematically using a socioecological framework.

**Results:**

Median prevalence of reported non-contact CSA was 57.0% (range: 31.1%–80.0%). Contact CSA prevalence was higher among females (median: 14.9%, range: 2.2%–40.2%) than males (median: 6.0%, range: 0.6%–21.2%). Regional variations were notable, with sub-Saharan Africa reporting the highest contact CSA prevalence (median: 25.5%) and Latin America and the Caribbean the lowest (median: 8.5%). Vulnerable groups, including Indigenous children (median: 39.2%), orphans (37.9%) and the incarcerated (31.1%), faced higher risk. Risk factors spanned individual (shame, stigma), familial (poor supervision, dysfunction), institutional (lack of safeguards) and societal (weak legal frameworks, normalisation of abuse) levels.

**Conclusions:**

Prevalence of CSA was globally high, with 14 in 100 females reporting having experienced CSA. Multilevel interventions, particularly strengthening family supervision and institutional safeguarding, legal accountability and community awareness, are critical for the prevention of CSA.

## Introduction

 A pervasive child health and safeguarding concern, child sexual abuse (CSA) negatively impacts children’s psychosocial development.^1^ While the definition of CSA varies considerably across the literature,^2 3^ the WHO defines CSA as ‘the involvement of a child in sexual activity that he or she does not fully comprehend, is unable to give informed consent to, or for which the child is not developmentally prepared, or else that violates the laws or social taboos of society. Children can be sexually abused by both adults and other children who are—by virtue of their age or stage of development—in a position of responsibility, trust or power over the victim’.^4^ Consistent with this definition, the literature generally recognises three forms of CSA: (1) non-contact abuse, including online abuse that exposes children to sexual activity that does not involve physical contact; (2) contact abuse that, for example, can include sexually intended touch and (3) penetrating abuse to children, for example, physical sexual penetration.^5^

Feelings of guilt, embarrassment and shame, intertwined with sociocultural taboos and stigma surrounding sexual assault, all contribute to global underreporting of CSA.^6^ The immediate and lifelong sequelae, however, are well-documented and far-reaching.^7^ Survivors may experience cognitive and emotional disturbances, including anxiety, depression, suicidality and suicide attempts, and post-traumatic stress disorder.^8^ CSA is also linked to disruptions in academic achievement, future economic earnings, impaired social relationships, increased risk-taking behaviours and long-term physical health complications such as chronic pain, substance abuse, revictimisation and cardiovascular disease.^9^ Beyond the individual level, CSA has broader societal implications, including stress on economy of healthcare, social welfare and criminal justice systems.^10^ Given these extensive and lasting consequences, effective prevention strategies, timely identification, comprehensive support services and access to justice for victims, as well as appropriate legal consequences for perpetrators, are critical to mitigating the impact of CSA.^11^ These consequences underscore the critical role of paediatric, mental health and child protection services in early identification, prevention and long-term support for affected children.

CSA is associated with several risk and protective factors.^2 3^ Common risk factors include time spent by children in institutional settings, sex (with females at greater risk), younger age, disability, socioeconomic vulnerabilities, drug and alcohol use, and poor parental guidance, supervision and safeguarding policies.^12^ In addition, several factors are identified as inhibiting or promoting timely CSA disclosure, including access to supportive and protective services; sociodemographic variables such as age, gender, minoritised ethnic status; the failure to recognise disclosure as an iterative, interactive process; sociocultural norms, including norms around ‘child safety’; family cohesion; and experiences of shame.^13^ Understanding these factors is essential for informing context-appropriate screening, safeguarding practices and prevention strategies across child health systems.

Several systematic reviews have explored the burden of CSA, frequently focusing on specific countries or regions, or concerning children with specific characteristics (eg, homelessness, psychological and/or psychiatric disorders and disability), and are thus often constrained by the sample or context of the study.^5 6^ Few have explored the mix of prevalence of CSA and its adverse consequences.^5^ A report by UNICEF acknowledges a wide variation in the prevalence of CSA across different population groups and geographic regions, with the prevalence ranging from 7% to 12% in boys, 18% to 20% in girls and a greater prevalence among vulnerable populations worldwide.^3^ Meta-analyses on the prevalence of CSA are often limited by methodological heterogeneity, contextual variability and self-reporting biases, which warrant a more inclusive approach characteristic of a scoping review.^14 15^ Moreover, previous global prevalence meta-analyses highlight an incongruence between official reporting rates and the self-declared rates reported in prevalence studies.^14 15^ The latter suggests the presence of multiple barriers that prevent CSA disclosure, as an important step to mitigate the impact of CSA on health and mental well-being of children and prevent further victimisation. The main objective of this study was to synthesise and map global evidence on the prevalence of CSA across abuse types, sexes, regions and vulnerable populations, and to examine the key individual, familial, institutional and societal risk factors contributing to its occurrence using a socioecological framework.

## Materials and methods

### Design

This was a systematic literature review adapted to comprehensively capture the scope and breadth of evidence on CSA including drivers using a socioecological lens. The review was also designed to accommodate the heterogeneity of studies and map their findings (online supplemental file S1). The review and/or its protocol have not been published or registered.

### Search strategy

Search strategies following the tenet of systematic literature review were developed to identify relevant articles across three major databases: PubMed, Scopus and Web of Science. Search terms included keywords related to ‘child sexual assault’ and ‘prevalence’ with a sample search string, adapted for three major databases, provided in online supplemental file S2. After removing duplicate publications, titles and abstracts were screened to identify potentially eligible articles by BA, AD and AM. The review was conducted and reported in accordance with the Preferred Reporting Items for Systematic Reviews and Meta-Analyses guidelines (online supplemental file S3). Articles selected based on this initial screening were then read in full text against the full eligibility criteria (table 1). Where full texts were unavailable, the authors attempted to contact corresponding authors and relevant institutional libraries.

**Table 1:** Inclusion and exclusion criteria

Inclusion criteria	Exclusion criteria
Relevance to CSA: studies that report data specific to child sexual abuse. Geographical scope: Any country. Combination with adult data: Studies reporting combined child and adult sexual abuse data were included only if CSA-specific prevalence could be disaggregated. Literature type: Published academic literature and grey literature. Type of study: all types of methodologies (eg, cross-sectional, longitudinal and combined methods) Studies reporting prevalence without restricting by time and period	Lack of specific CSA data: studies that reported child abuse or violence against children without specific information on CSA. Irrelevant data: studies that did not report prevalence of CSA. Studies reported in a language other than English.

CSA, child sexual abuse.

### Data extraction

Data were extracted from the selected articles using a piloted data extraction sheet. Apart from re-examining the relevance based on the reading of full text, articles were not subjected to a quality appraisal. This served our objective by being accommodative to a wide range of evidence—an essential element for socioecological analysis which uses qualitative methods of data synthesis. In addition, our flexible approach also allowed us to examine the discordance and concordance of evidence without categorising which may have improved the comprehensibility.

The data extraction procedure followed a regular meeting and discussion among the core team members. After extracting data from the first ten articles, core team members convened to adapt and discuss the methods and types of data to be extracted. Three authors (AD, AM and BA) independently extracted data into a Microsoft Excel sheet. A standardised Excel form was designed to extract the following essential information from included studies: country, year, sample (sex, age, sample size), study design, relevant laws related to CSA, prevalence of CSA, measure of CSA, facilitators and barriers.

Any discrepancies among the three authors during the extraction were resolved through regular meetings together with a fourth author (NS) who provided the additional input. A consensus-based approach was used. The final discussion involved re-examination of the full text and revision in the extracted data.

### Grey literature

In addition to academic literature, grey literature such as reports from UNICEF and government reports (eg, UK’s CSA reports) were searched using Google and Google Scholar. Suggestions from authors and experts in the field were also incorporated to broaden the inclusion of literature. Some of these institutions were contacted for relevant documents. Grey literature searches were conducted using predefined keywords, and documents were screened using the same eligibility criteria as peer-reviewed studies. A total of nine reports were reviewed, and their findings were incorporated into this review (online supplemental file S4).

### Data analysis

Included studies were grouped according to age group, World Bank country regions^16^ and priority population examined. Prevalence rates across regions and income levels were reported as a range (lowest and highest reported prevalence across studies). Median values and range from the prevalence across the categories were calculated. Given substantial methodological and contextual heterogeneity across studies, meta-analysis was not undertaken.

### Qualitative synthesis

Risk factors for CSA were examined through a qualitative analysis of the data. Three main domains for qualitative data extraction were: ‘background laws related to CSA’, ‘facilitators’, and ‘barriers’ related to CSA, and ‘authors’ reflection’. Textual data were first extracted at Microsoft Excel followed by coding at QSR NVivo using a mixture of inductive and deductive approaches applying a socioecological model (online supplemental file S5). Codes were later synthesised according to three categories of influence known to impact on general health outcomes, which, adapted from Bronfenbrenner’s socioecological framework, categorised data into macro, meso and microlevels.

- Microlevel: Individual-level risk factors, referring to a child or adolescent.
- Mesolevel: Family and institutional factors, including schools, colleges and training centres.
- Macrolevel: Societal and policy-level factors affecting CSA.

This approach allowed for a comprehensive understanding of the complex factors influencing CSA globally.

## Results

Following the search strategy, a total of 8086 articles were collated in the EndNote in August 2024. After removing the duplicates, a total of 5493 articles were screened based on the title and abstracts. Finally, a total of 398 full texts were assessed for eligibility, resulting in 332 articles for data analysis (online supplemental file S4). While the prevalence of CSA was extracted from all 332 articles, for the comparability across the specific variables differentiated by gender, a total of 154 articles were selected for median prevalence calculation. The prevalence of CSA varied across geography, age groups and population types. In general, non-contact CSA was higher (median: 57.0%, range 31.1%–80%;) than contact-based CSA (overall median: 14.1%, range 0.5%–60.4%;) with females at higher risk (median: 14.9%, range: 2.2%–40.2%) than males (median: 6%, range 0.6%–21.2%). The majority of the reported CSA incidents occurred within a familiar social space, where children spend most of their time. The perpetrators were predominantly family members or close relatives. The prevalence of CSA is likely to be underreported due to stigma, embarrassment and shame.

### Study characteristics

Of 383 studies selected for full text data analysis, 332 were primary studies and 51 were evidence syntheses. The data from the latter were excluded when analysing the prevalence data. The wide heterogeneity of study methodology, population and study instruments constrained the pooling of prevalence estimates including the breadth of the range reported. Finally, prevalence rates from a total of 154 articles were compared against each sub-group (online supplemental file S6). Diverse measures were used to calculate CSA prevalence, the most common of which are presented in box 1.

Box 1 **Common tools used to measure child sexual abuse in the literature**

1. International Society for the Prevention of Child Abuse and Neglect (ISPCAN) Child Abuse Screening Tools Retrospective version (ICAST-R).
2. Childhood Trauma Questionnaire-Short Form.
3. Childhood Experiences of Care and Abuse Questionnaire.
4. Finkelhor’s (1979) questionnaire for measuring sexual abuse.
5. Adverse Childhood Experiences International Questionnaire.
6. Ten-item Kessler Psychological Distress Scale.
7. IISPCAN ICAST-R and Child Abuse Screening Tool Children’s Version.
8. Childhood Abuse and Neglect Questionnaire.
9. Child Abuse Experiences and Sexual Harassment scale.

Some studies combined measures of CSA with other psychological trauma assessment scales, disability assessments, substance and alcohol abuse; for example, the WHO’s quality of life scale, the Centre for Epidemiologic Studies Depression Scale and Rosenberg Self-Esteem Scale.

Studies reporting prevalence of CSA (n=332) were further separated based on whether the CSA prevalence specific to gender (eg, male and female) was reported. Among the studies which reported prevalence by gender (n=154), 46 included children or adolescents as respondents. In another 11 studies, the respondents were a combination of children and adolescents. Forty-two of 154 studies were from North America (27%), followed by Europe and central Asia (n=35; 23%), East Asia and Pacific (n=31; 20%). Among the 49 studies investigating subpopulations, 11 studies interviewed psychiatric patients, men who have sex with men (n=10), and low-income and middle-income families (n=9).

### Prevalence of CSA

**Figure 1:**
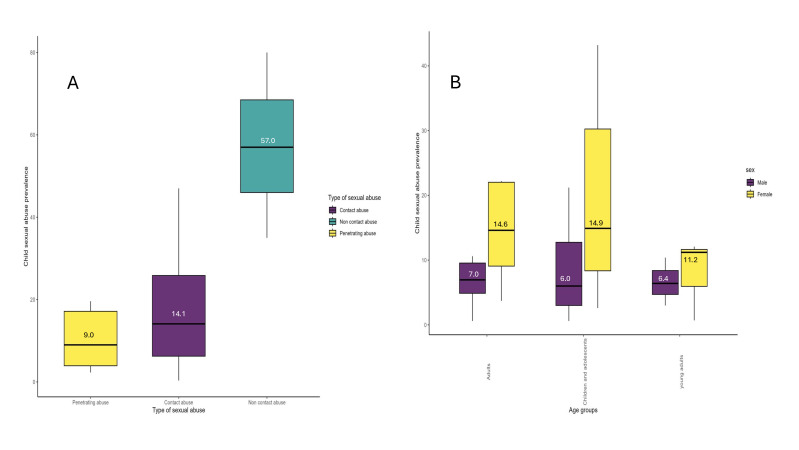
Prevalence of reported child sex abuse. (A) Median prevalence of reported child sex abuse by category of abuse; (B) Median prevalence by sex and age group.

The median prevalence of reported non-contact CSA was higher than that of contact and penetrative abuse (figure 1A), with median values of 57.0% for non-contact abuse, 14.1% for contact abuse and 9.0% for penetrative abuse.

Contact CSA was further disaggregated by age group of the respondent, including adults aged 25 years and older, children and adolescents under 19 years, and young adults aged 20–24 years (figure 1B). The median prevalence for these groups was as follows: children and adolescents under 19 years reported a median prevalence of 6.0% for males and 14.9% for females, young adults aged 20–24 years reported a median prevalence of 6.4% for males and 11.2% for females, and adult respondents aged 25 years and older reported a median prevalence of 6.9% for males and 14.6% for females.

**Figure 2:**
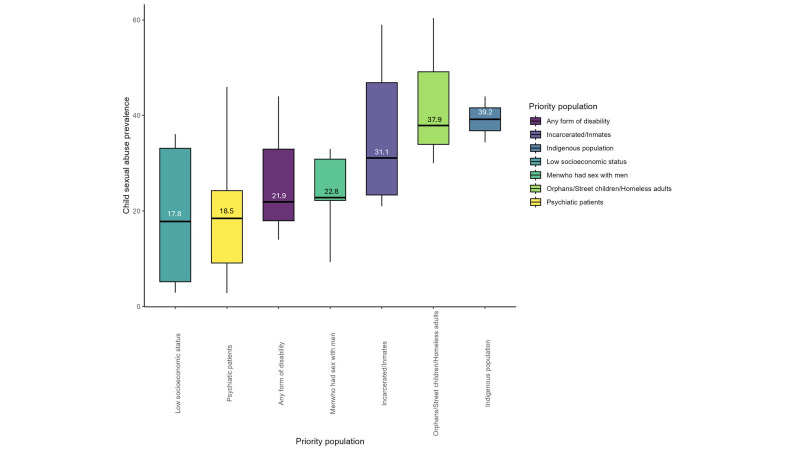
Prevalence of reported child sexual abuse. Median prevalence by priority population.

**Figure 3:**
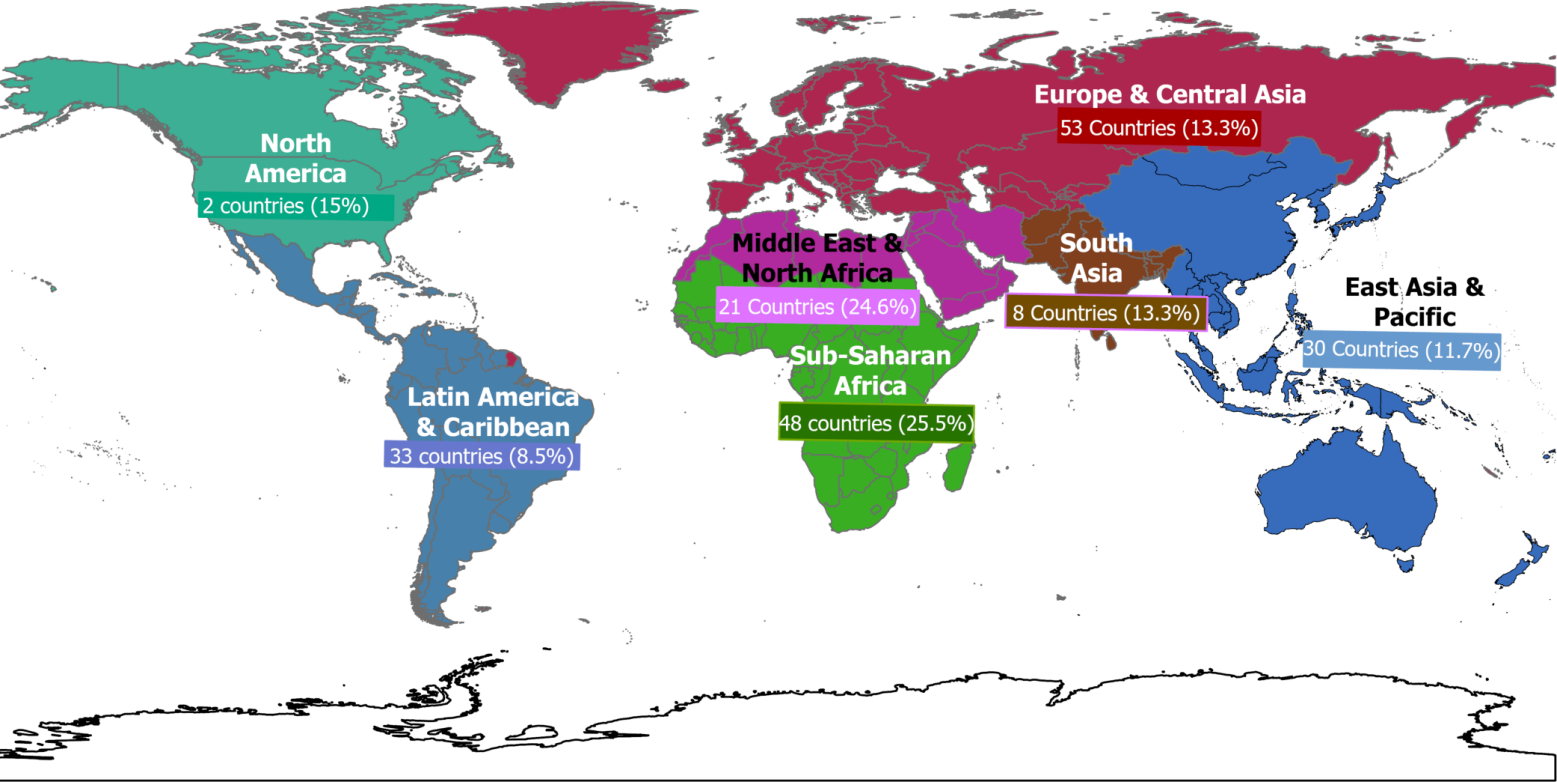
Prevalence of reported child sexual abuse by world bank regions.

The reported prevalence of CSA was highest among Indigenous populations, with a median prevalence of 39.2%, followed by those who were orphaned or homeless, with a median prevalence of 37.9% (figure 2). When disaggregated by World Bank regions, Western and Central Africa had the highest reported median prevalence of 25.7%, compared with the lowest CSA in Latin America and the Caribbean at 8.5% (figure 3).

### Risk factors associated with CSA

**Figure 4:**
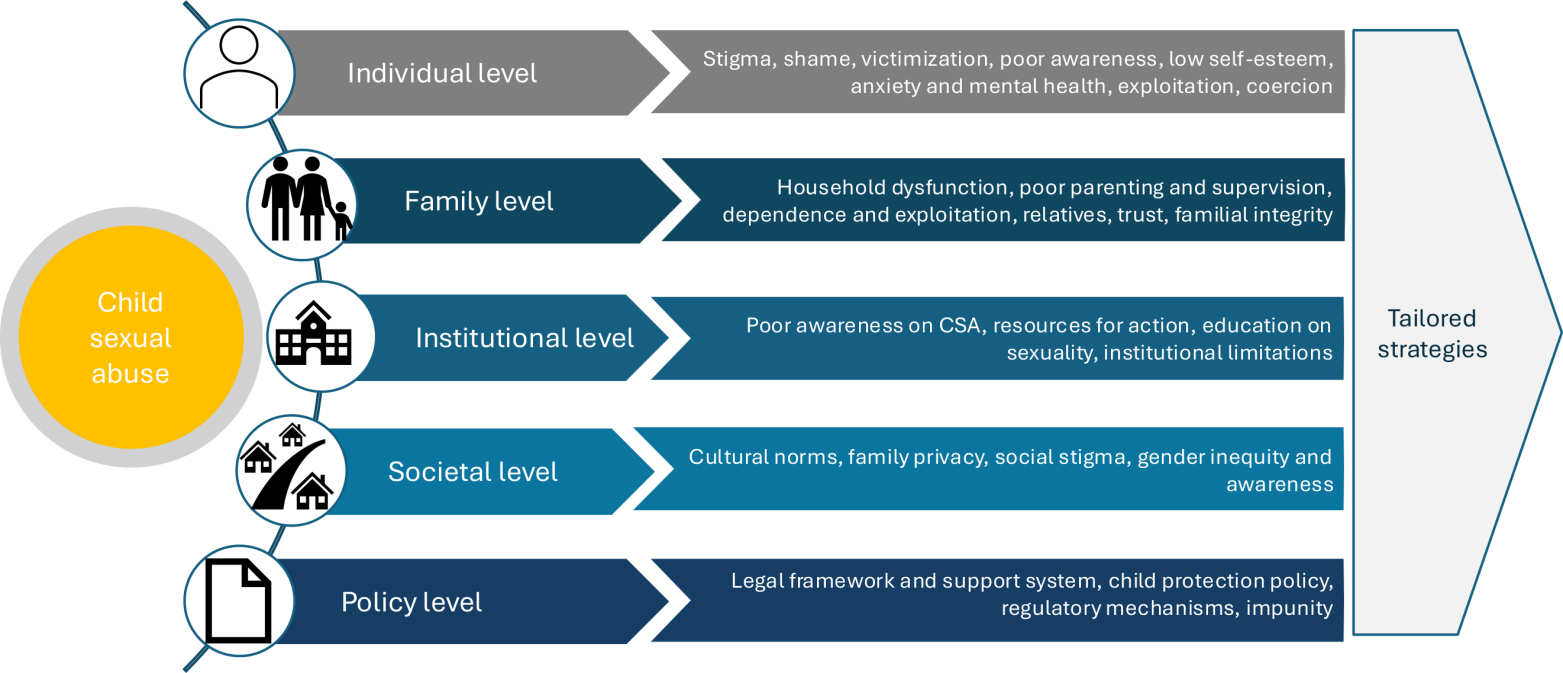
Factors affecting child sexual abuse at various levels based on the literature. CSA, child sexual abuse.

Risk factors contributing to CSA were extracted from the literature (online supplemental file S7). Risk factors of CSA overlapped across the categories. The higher recurrence of risk factors was embedded in family-level characteristics, followed by societal, individual and policy characteristics of the country (figure 4).

### Individual level

At an individual level, victims were frequently found to hesitate to report and seek help because of stigma, shame and the process of victimisation. The victims of CSA often anticipated disbelief, shame and potential repercussions from family or community. The self-afflicting nature of stigma when reporting or disclosing sexual abuse was a critical hindrance to reporting. The process of victimisation often entailed shame and embarrassment while incurring continued abuse or even re-victimisation. Female children were reported to be more likely to experience mental health issues, increasing their vulnerability to CSA, compared with their male counterparts. Few studies reported no difference in CSA by gender. Others reported increased vulnerability in male children. Children, particularly younger ones, may well be unaware of what constitutes CSA, when to seek help and how to describe the acts of abuse they have undergone. CSA victims were likely to suffer psycho-social trauma of abuse, lowered self-esteem, anxiety and other mental health conditions that complicate and delay reporting these issues. At the same time, psychological conditions such as childhood attachment challenges (eg, insecure attachment) and emotional regulation difficulties were associated with increased vulnerability to CSA. Children may be exploited in power-imbalanced relationships with those who they trust most, such as adults (caregivers) and relatives. Similarly, disability, substance abuse, poverty and absence of a supportive environment were found to increase the risk of CSA.

### Family level

Family-level factors were found to be the predominant risk for CSA. Specifically, dysfunctional households, poor parenting and inadequate supervision were major contributors. Dysfunctional households included divorced parents, single parents, disputes within the family, domestic violence, separated parents and poor cohesion within the family members. Presence of a stepfather, foster parents and male cousins also affected the risk of CSA. Dysfunctional parenting was often linked to poor supervision of children, whereby lack of care, affection and monitoring of children’s behaviour added vulnerability to CSA.

Children not reared by their biological parents were found to be at increased risk amidst their non-biological parents, including foster parents, as well as relatives and peers. Children in care and dependent on guardians were vulnerable to exploitation by caregivers. Most literature alluded to such exploitation by close relatives, who exploited the close proximity and intimacy in familial relationships. Under such circumstances, sexual abuse was difficult to report, because familial integrity and relationships seemed to be given greater priority than prevention and punishment of sex abuse.

Children of parents misusing alcohol or other mind-altering substances were at increased risk of child sexual exploitation. Mental health conditions in parents were additional risk factors for CSA. When a father or mother had a mental illness, children were more likely to face negative outcomes due to inadequate supervision and disruptive or harmful behaviours towards them.

### Institutional level

Institutions such as schools, colleges and any other places where children spend extended periods of time can become a social space where children are vulnerable to CSA. Reported risk factors include a lack of awareness by the victim of what constitutes CSA, how it occurs and how to tackle it. This was particularly relevant within institutions where resources for the prevention of CSA were limited or absent, such as church-sponsored boarding schools. Most articles highlighted a lack of sex education and other training on topics related to sexuality, sexual development and the identification of inappropriate sexual behaviour as risk factors.

Some studies reported the exploitation of trust and power imbalance between teachers/trainers and their students. In such scenarios, the reporting of abuse and action against the perpetrator can reach an insurmountable level of complexity and intricacy. When institutions were ready to tackle cases of CSA, they were often restricted by the nature of CSA (non-contact based sexual harassments, apparently considered as normal behaviour). The bureaucratic complexity required by regulatory agencies, laws and the need for parental involvement frequently led to the evasion of CSA cases.

### Societal level

A significant number of studies identified the social and cultural norms that prevent CSA victims from disclosing, reporting and taking action against their perpetrators. Many of these studies were located inlow- and middle- income countries (LMICs), where social and cultural norms banned or restricted conversations about CSA. Contexts where cases are commonly stigmatised in society were also viewed as barriers to disclosure.

Inequity in gender and power imbalances, particularly in patriarchal societies, was found to hinder fair discussion and legal action. In such contexts, female victims face an added layer of complexity when attempting to report CSA. Lack of social support was a major hindrance to disclosing, reporting and taking actions against CSA, compounded by higher presence of perpetrators in some communities.

### Policy level

A significant number of articles reported the presence of a general child protection law and regulation that encompassed the sexual abuse, while specific CSA-related measures were either underreported or were simply absent. Overall, however, the lack of a legal framework and absence of accompanying support systems were seen as primary policy-level barriers to mitigate the risk of CSA. The absence of safeguards to prevent CSA is a major barrier to the legal processing of perpetrators, not only by punishment but also in terms of preventing future sexual abuse. Impunity also played a significant role, allowing such cases to evade justice. The absence of a specific policy on CSA, or poor regulatory implementation, was another major barrier. Authorities at times intentionally evaded the prosecution of CSA cases when they had conflicting interests, and in doing so, eroded the trust, power and relationships invested in them.

## Discussion

The global prevalence of CSA was found to be high and varied by population types, region, age group and the instruments used. Non-contact CSA ranged from 31.1% to 80.0% (median: 57.0%) and contact-based CSA varied by sex and age groups (children/adolescents; males: median: 6.0%, range 0.6%–21.2%; females: median: 14.9%; 2.2%–40.2%,), region and subpopulations (higher among indigenous population: 14.0%–50.0%; and disability affected population: 21.9%–42.4%). The heterogeneity in CSA by sex, region and disability is echoed by previous, although narrower reviews.^56 17^,^25^ Although the heterogeneity in prevalence varied by sociodemographics, region and health conditions, literature widely acknowledges the significant underreporting of CSA. Some studies reported that only 10% of incidents resulted in formal complaints, undermining global efforts to understand true population prevalence.^26^ The disclosure from the victims and prevention of recidivism is heavily inhibited by the stigma attached to such incidents.^3 27^

The variability in CSA prevalence may reflect the complex interplay of factors across multiple social spheres, including individual circumstances, familial dynamics, institutional structures, community environments and broader policy contexts.^28^ The most salient and common feature of such incidents across the geographies, social and cultural context is the stigma that inhibits disclosure, that acts as a barrier to prosecuting perpetrators.^24^ While reporting may bring perpetrators into legal proceedings, it may disproportionately traumatised the victim, through negative self-representation, or failure to obtain justice, leading to unintended repercussions.^25^ In many settings, legal procedures cannot assure the timely and permanent separation of victim and perpetrator.

Another common feature of CSA was how often such incidents occurred within a close living space, shared by family members and close relatives. This finding corroborates an independent inquiry on CSA conducted in the UK. More than unacquainted outsiders, CSA occurred often within the close family space, when trust and relationships were exploited by family members or relatives. Intrafamilial CSA incidents are often kept secret to maintain familial integrity.^29^ The emerging concerns in such scenarios across the literature reflect poor parental supervision and monitoring of children. It has been established that poor parental supervision can increase the risk of children’s offending and avoidant attachment, resulting in increased vulnerability to CSA and further inhibiting disclosure.^30^

Educational institutions are another social sphere where children and adolescents spend time.^31^ Institutions with poor supervision and monitoring of their students, for example, interactions and relationship among peers, and between students and their teachers, can have a major bearing on how an institutional environment, also referred to as ‘institutional risk’ that can predispose to CSA.^32^ Adverse interactions and abusive behaviours were further affected by institutional norms, regulations and support services. Lack of awareness of what CSA is, its prevalence, varying types and severity of infliction, and the availability of specific services to prevent, mitigate and punish such incidents was found to be limited, particularly so in LMICs.^33^

CSA seems to flourish in a social environment where stigma is high and victims are ashamed and silent.^34 35^ Cultural and social stigma affected risk of CSA in two ways. First, it hindered the disclosure by the victim, and second, it condoned, trivialised and ultimately created circumstances for the crime(s) to continue.^36^ These social characteristics meant that there was often a lack of social support and services for victims, fostering repeated CSA and immunity for perpetrators.

Continued exposure to the perpetrator was also found to discourage reporting of the crime.^25^ The inability of social services and law enforcement to separate the victim from the perpetrator reduced the effectiveness of punitive action against the perpetrators.^37^ Although the presence of child protection laws and regulations existed to support the victims, the literature reported little impact of such legal frameworks, particularly in LMICs. For instance, in Indonesia, such laws suffered from unclear conceptualisation of what constituted CSA, further compounded by the societal norms.^38^ The poor prospect of meaningful support, specifically not having to face the perpetrator again, may therefore be more important than the specific provisions outlined in laws or constitutions.^39^ Stringent policies in many LMICs are often more symbolic than functional—well-articulated but ultimately unenforceable.

### Implications for risk mitigation and prevention of CSA

This review was prompted by reports of CSA during a community randomised controlled trial evaluating a novel housing intervention in southern Tanzania, leading investigators to explore the issue more deeply.^40^ To understand the scope and impact of CSA, a review of global literature was conducted to inform future research, support affected individuals and field staff and guide the design of preventive interventions.

Consistent with the previous literature, the review offers three main conclusions. First, current estimates of CSA prevalence are heterogenous owing to methodological and contextual differences. Second, a multitude of factors inherent at various social levels contribute to CSA. Finally, CSA is likely to be highly underreported given concerns related to disclosure, stigma and consequences. The fundamental steps to tackle CSA include increasing the awareness and engagement among stakeholders such as community members, policy makers and local authorities responsible for community welfare. Destigmatisation campaigns to promote prompt reporting and action are critical, yet calls for disclosure must also occur within reliable and implementable policies and legal recourse for perpetrators. Prevention of CSA further requires separation of the victim and the perpetrator, which is only possible if there is a safe place for the victim and the successful implementation of legal procedures, including, at a very minimum, a restraining order placed against the perpetrator. Only if community members realise that the victims receive protection will other victims come forward, and perpetrators will be held accountable.

### Strengths and limitations

The main strengths of this review are the breadth of literature included to draw the global prevalence of CSA. The review follows a standard search strategy employed in systematic literature reviews and deviates only during data extraction, insofar as necessary to ensure the inclusion of all forms of evidence, while also allowing for qualitative synthesis of the data. Ensuring inclusivity, without appraisal of the quality of evidence, may have compromised the confidence. Within the literature on CSA prevalence, we explored contextual characteristics where they occur, including factors affecting their occurrence. Analysis of risk factors within the CSA prevalence literature allowed us to extract risk factors that may have a direct link to prevalence. However, such qualitative factors may be secondary in studies primarily focusing on the prevalence of CSA. The studies under review included a wide range of scales to assess the prevalence of CSA, some specifically to measure CSA, others combined with psychological assessments and self-constructed survey instruments. The heterogeneity in the instruments, methodology and contexts of study restricted our confidence to pool the prevalence. Few studies reported age standardised prevalence of CSA. That said, variance in the reporting and specifying the characteristics of the population was a major challenge to establish reliable global estimates. Since we are working with summary data, not the individual participant data, we cannot report age standardised prevalence; instead, we report three groups of respondents: children and adolescents, young and older adults. Despite the heterogeneity of the prevalence across the population groups, age groups and geographical regions, the estimates in our review corroborate the ubiquity of CSA across cultures and contexts.

## Conclusions

The occurrence of CSA is shaped by layers of social spheres, where children occupy the same environments as adults. Because of a high level of stigma, CSA often remains undisclosed and thus affects our ability to determine its true prevalence globally. The prevalence estimates in our report must therefore be understood as minimum estimates. Definitive steps to prevent repeated CSA are often complex, particularly in LMICs, where safe places for victims can be difficult to establish. Layered approaches to CSA are essential, including engaging with the family and community for social support.

## Supplementary material

10.1136/bmjpo-2025-004423 online supplemental file 1

10.1136/bmjpo-2025-004423 online supplemental file 2

10.1136/bmjpo-2025-004423 online supplemental file 3

10.1136/bmjpo-2025-004423 online supplemental file 4

10.1136/bmjpo-2025-004423 online supplemental file 5

10.1136/bmjpo-2025-004423 online supplemental file 6

10.1136/bmjpo-2025-004423 online supplemental file 7
