# Supplementary material for: Mapping the global prevalence and socioecological drivers of child sexual abuse: a systematic review and synthesis
Source: BMJ Paediatr Open. 2026 Apr 3;10(1):e004423. doi: 10.1136/bmjpo-2025-004423 (PMC13052705; doi:10.1136/bmjpo-2025-004423)
Supplement: online supplemental file 2 [file bmjpo-10-1-s002.pdf]

Date of last search 27 August 2024

(1) (sex* AND (abus* OR assault*))

(2) AND (child* OR adolescen* OR infant* OR preschool OR “school age” OR teen*)

(3) AND (Epidemiology OR prevalence [MeSH Terms] OR prevalence)

Pubmed (n=2126)

Scopus (n=3371)

Web of Science (n=2589)
