# Supplementary figures and images for: Mapping the global prevalence and socioecological drivers of child sexual abuse: a systematic review and synthesis
Source: BMJ Paediatr Open. 2026 Apr 3;10(1):e004423. doi: 10.1136/bmjpo-2025-004423 (PMC13052705; doi:10.1136/bmjpo-2025-004423)

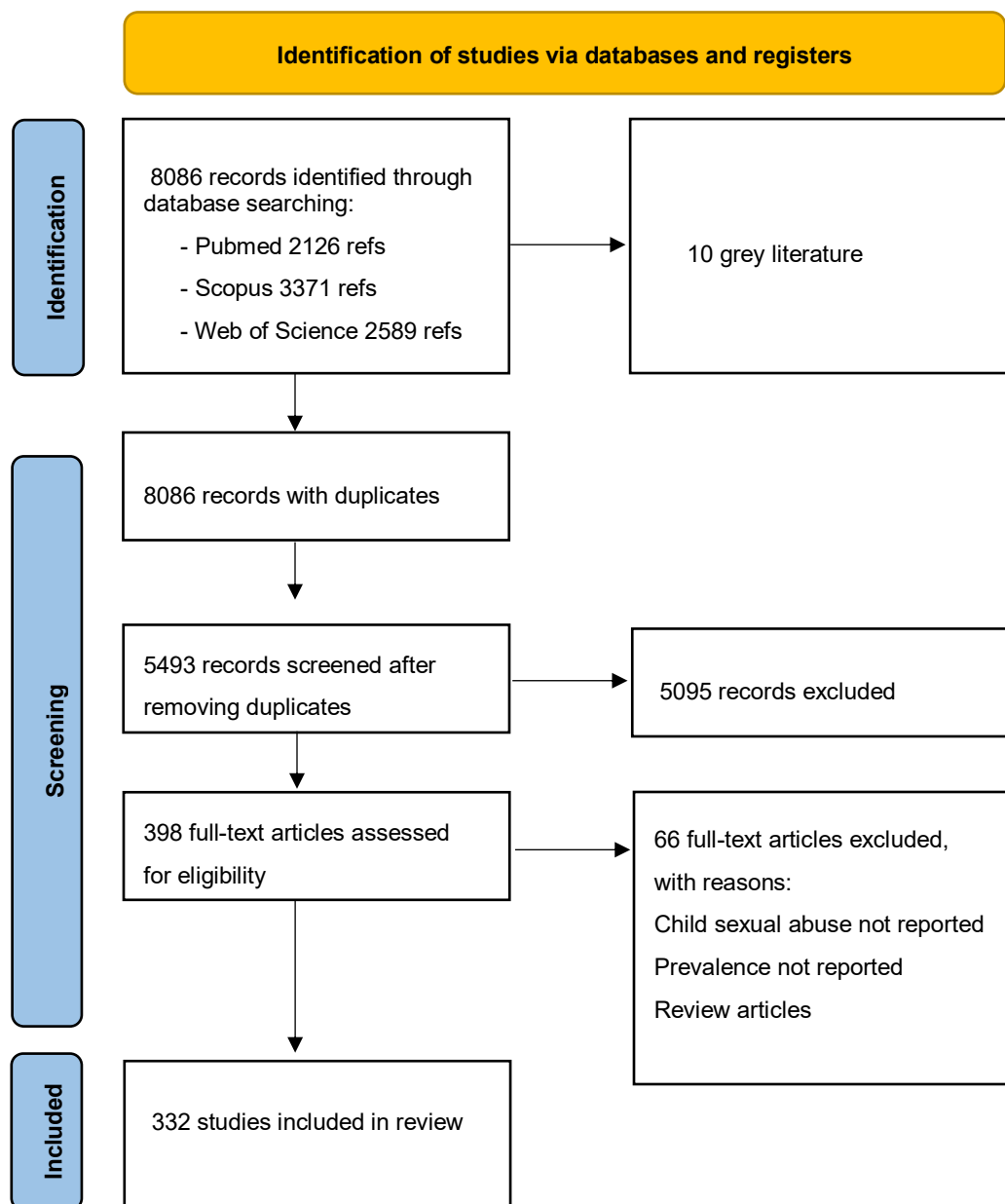

Figure 1: PRISMA flow diagram of the literature

Supplement: online supplemental file 3 [file bmjpo-10-1-s003.pdf]
