# Supplementary material for: Mapping the global prevalence and socioecological drivers of child sexual abuse: a systematic review and synthesis
Source: BMJ Paediatr Open. 2026 Apr 3;10(1):e004423. doi: 10.1136/bmjpo-2025-004423 (PMC13052705; doi:10.1136/bmjpo-2025-004423)

Supporting Information S5 File: Coding and references

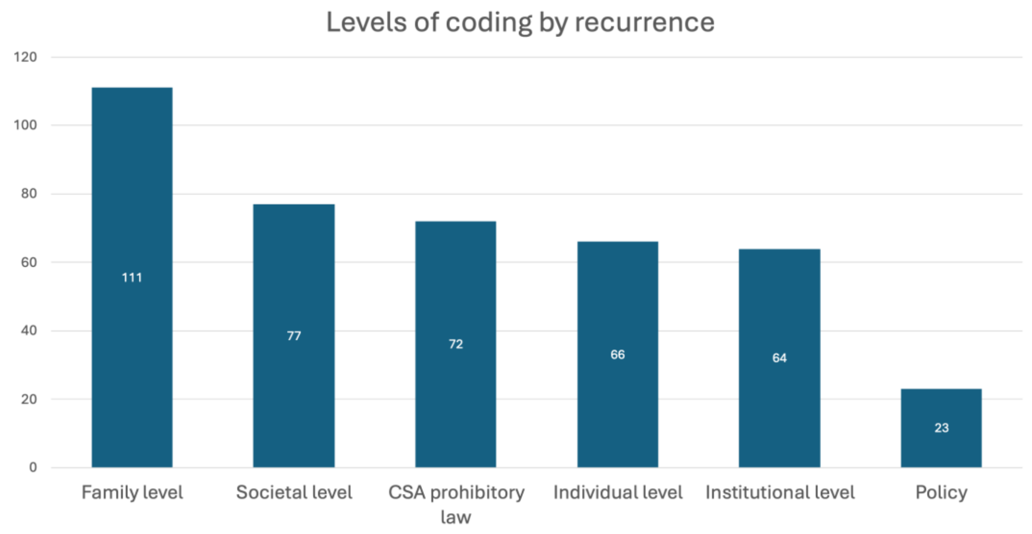

Codes

Name	Description	Sources	References
CSA prohibitory law		1	72
General child protection	Laws and regulations general to children's welfare.	1	39
Specific to sexual abuse	Laws and regulations specific to CSA.	1	33
Family level	Family level factors	1	111
Alcohol, mental health and substance abuse by parents	Alcohol and substance abuse within the family increases risk behaviour.	1	14
Awareness	Lack of awareness and understanding of the issue among caregivers and the community.	1	10
Dependence and exploitation	Dependence to caregivers, grooming, exploitation of trust and relationships.	1	21
Household dysfunction	Divided family, quarrel, violence, separated, single parents.	1	37
Parenting and supervision	Parenting and supervision of child in terms of safeguarding the risks for CSA	1	32
Poverty	Poor socio-economic status affecting the vulnerability.	1	10
Power imbalance	Power imbalances among family members, relatives	1	7
Relatives, familial integrity	Perpetrator from close family relatives including the tension to maintain the familial integrity	1	19
Shame and fear of	Fear of repercussions, shame, and the potential for	1	9

Name	Description	Sources	References
repercussions	familial disruption.		
Individual level	Individual level predisposition. Factors inherent in an individual	1	66
Adverse childhood experiences	Presence of adverse childhood experiences, such as physical and emotional abuse.	1	1
Awareness	Awareness related to what CSA is and how to respond/report.	1	13
Coping strategies	Individual's coping strategies to CSA	1	1
Disability	Any form of disabilities affecting CSA	1	2
Exploitation coercion and trust	In situations where trust and power imbalances are present can allow exploitation, manipulation and coercion.	1	10
Peer effects	Peer's influence on adverse behaviour.	1	2
Poverty	Poverty induced vulnerability.	1	2
Relatives kinship	Relatives such as closed ones including those with close kinship are in position to exploit the relationship	1	3
Self-esteem, anxiety and mental health	Range of personality disorders including low self-esteem, anxiety and mental health problems	1	10
Stigma shame and victimization	Victims' fear of disbelief, shame, and potential repercussions from family or community	1	18
Substance abuse	Substance (e.g. alcohol, drugs, Khat) abuse by children predisposes to CSA	1	7
Support services	Support services available to children to understand, act and tackle the cases of CSA	1	2
Institutional level	Institutions such as schools, community, prison	1	64
Awareness	Awareness related to what CSA is, how to respond and resources to tackle the cases of abuses.	1	25
Complex institutional procedure	Complex and inefficient institutional procedure to respond to the CSA.	1	1
Education institutes' limitations	Lack of safeguarding policy in schools, resources, and action against CSA	1	14
Education on sexuality	Availability of sex education to students in formal institutions.	1	15
Exploitation of trust by authorities	Exploitation of trust by authorities (teachers, authorities, seniors) towards children.	1	9
Frequency of CSA	Prior history of CSA including number of times they have had such adverse experience is likely to affect their predisposition later.	1	2
Peers and friends	Impact of peers and friends predisposing to CSA risk behaviour	1	2
Resources for action	Resources for action to tackle CSA for e.g. human, guiding documents, education, training.	1	17

Name	Description	Sources	References
Policy	State and policy level factors	1	23
Child protection policy	Policy safeguarding child protection. Availability of such a policy.	1	2
Impunity	Lack of enforcement of regulatory frameworks, evasion of punishment. Loose regulatory implementation	1	5
Legal framework and support system	The presence of legal framework to act against the CSA. The presence of support system.	1	19
Prior history of CSA	History of CSA during their childhood. Prior history was seen to predispose them later in their adulthood.	1	1
Trust towards authority	Trust and distrust towards authority members regarding CSA as a problem.	1	2
Societal level		1	77
Awareness	Awareness about CSA, what it is and how it should be defined, detection, mitigation and action against it.	1	8
Cultural, Family privacy	Cultural taboos, cultural acceptance of CSA, social minimization of the CSA as an issue, trivialization, Social response, Familial tendency to keep the CSA incidents private at the familial level	1	52
Gender and inequity	Disproportionate burden of CSA by gender	1	9
Grooming	Any grooming activities that blurs the care and affection	1	1
Perpetrators	High presence of perpetrators in the society	1	1
Social stigma	Stigma perceived by an individual towards CSA in their society	1	21
Social support	Socially available support system or mechanisms.	1	7

Child sexual abuse-systematic review (Individual level)

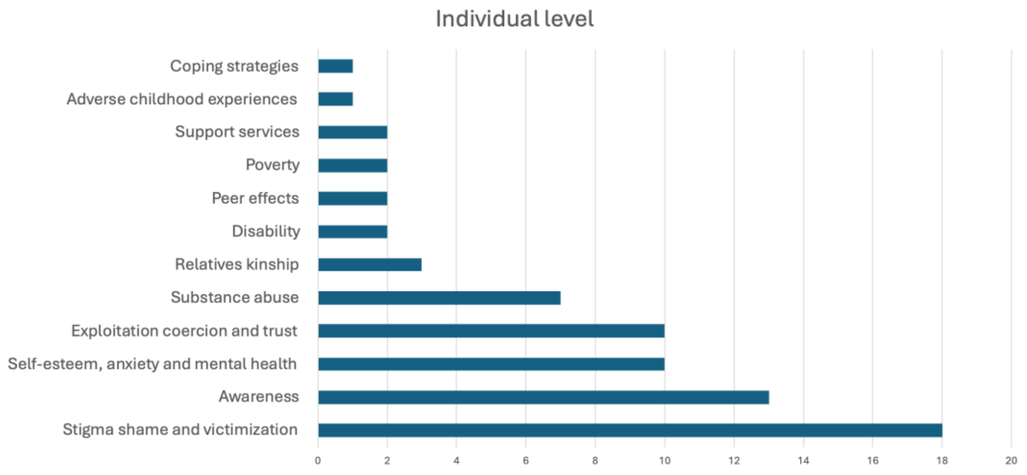

Child sexual abuse-systematic review (Family level)

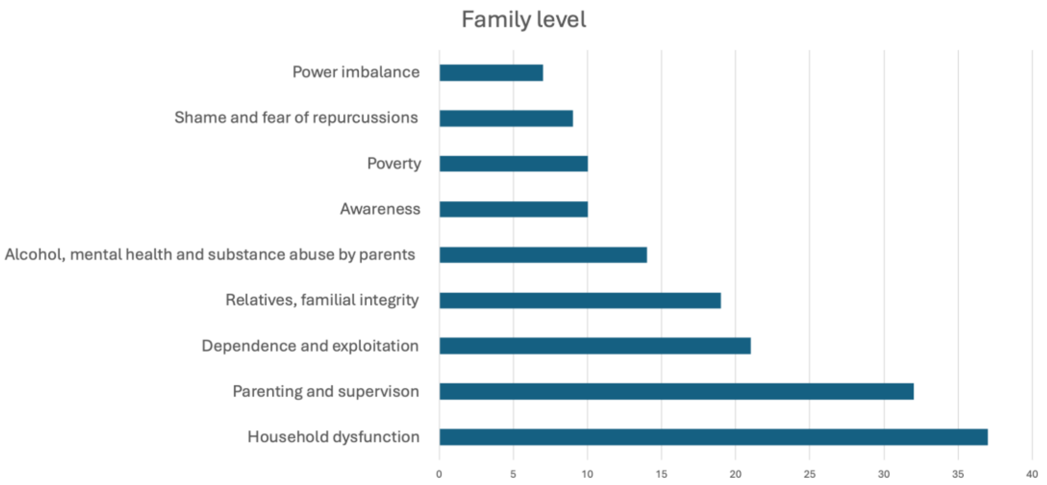

Child sexual abuse-systematic review (Institutional level)

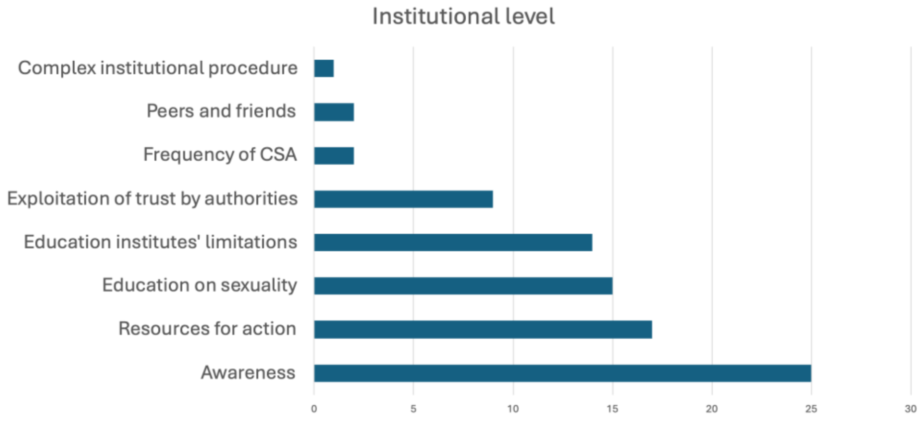

Child sexual abuse-systematic review (Societal level)

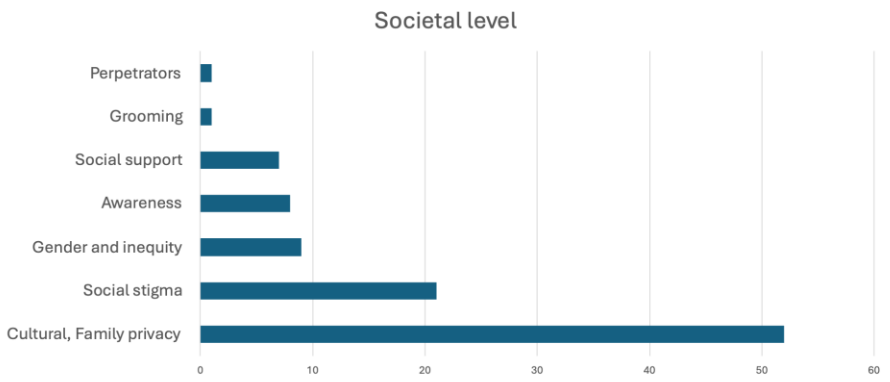

Child sexual abuse-systematic review (Policy level)

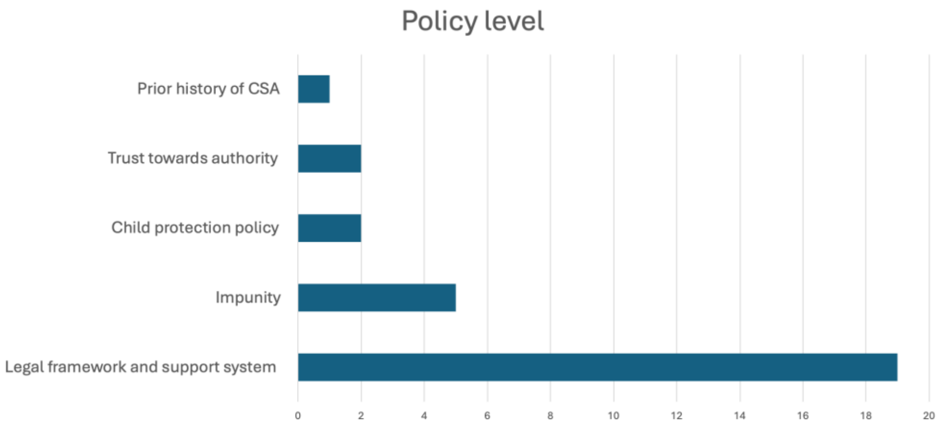

Child sexual abuse-systematic review (Background law)

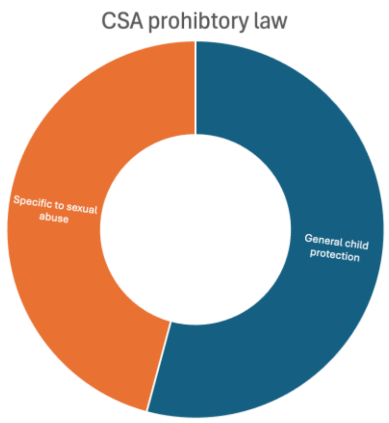

Supplement: online supplemental file 5 [file bmjpo-10-1-s005.pdf]
