# Supplementary material for: Mapping the global prevalence and socioecological drivers of child sexual abuse: a systematic review and synthesis
Source: BMJ Paediatr Open. 2026 Apr 3;10(1):e004423. doi: 10.1136/bmjpo-2025-004423 (PMC13052705; doi:10.1136/bmjpo-2025-004423)
Supplement: online supplemental file 7 [file bmjpo-10-1-s007.pdf]

Supplementary File 7

List of additional references

1. Almeida TC, Barreiros I. Online grooming among Portuguese adolescents and the COVID-19 lockdown: Relationship with other types of victimization. *CHILDREN AND YOUTH SERVICES REVIEW* 2024; **156**.

2. Blancaflor E, Balita LBS, Subaan VRS, Torres JADF, Vasquez KJP. Implications on the Prevalence of Online Sexual Exploitation of Children (OSEC) in the Philippines: A Cybersecurity Literature Review. 2022 5th International Conference on Computing and Big Data, ICCBD 2022; 2022; 2022. p. 34-8.

3. Karkoskova S, Ropovik I. The Prevalence of Child Sexual Abuse among Slovak Late Adolescents. *Journal of child sexual abuse* 2019; **28**(4): 452-71.

4. Aboul-Hagag KES, Hamed AF. Prevalence and pattern of child sexual abuse reported by cross sectional study among the university students, sohag university, Egypt. *Egyptian Journal of Forensic Sciences* 2012; **2**(3): 89-96.

5. Halpérin DS, Bouvier P, Jaffé PD, et al. Prevalence of child sexual abuse among adolescents in Geneva: Results of a cross sectional survey. *British Medical Journal* 1996; **312**(7042): 1326-9.

6. Hébert M, Tourigny M, Cyr M, McDuff P, Joly J. Prevalence of childhood sexual abuse and timing of disclosure in a representative sample of adults from Quebec. *Canadian Journal of Psychiatry* 2009; **54**(9): 631-6.

7. Korkman J, Antfolk J, Fagerlund M, Santtila P. The prevalence of unfounded suspicions of child sexual abuse in Finland. *Nordic Psychology* 2019; **71**(1): 39-50.

8. Krause KH, DeGue S, Kilmer G, Niolon PH. Prevalence and Correlates of Non-Dating Sexual Violence, Sexual Dating Violence, and Physical Dating Violence Victimization among U.S. High School Students during the COVID-19 Pandemic: Adolescent Behaviors and Experiences Survey, United States, 2021. *Journal of interpersonal violence* 2023; **38**(9-10): 6961-84.

9. Levine E. Sexual Violence Among Middle School Students: The Effects of Gender and Dating Experience. *Journal of interpersonal violence* 2017; **32**(14): 2059-82.

10. Lodico MA, Gruber E, DiClemente RJ. Childhood sexual abuse and coercive sex among school-based adolescents in a midwestern state. *The Journal of adolescent health : official publication of the Society for Adolescent Medicine* 1996; **18**(3): 211-7.

11. Moore EE, Romaniuk H, Olsson CA, Jayasinghe Y, Carlin JB, Patton GC. The prevalence of childhood sexual abuse and adolescent unwanted sexual contact among boys and girls living in Victoria, Australia. *Child Abuse and Neglect* 2010; **34**(5): 379-85.

12. Pineda D, Muris P, Martinez-Martinez A, Piqueras JA. Prevalence of Child Sexual Abuse in Spain: A Survey Study. *EUROPEAN JOURNAL OF PSYCHOLOGY APPLIED TO LEGAL CONTEXT* 2023; **15**(2): 83-8.

13. Sariola H, Uutela A. The prevalence of child sexual abuse in Finland. *Child Abuse and Neglect* 1994; **18**(10): 827-35.

14. Vives-Cases C, Sanz-Barbero B, Ayala A, et al. Dating Violence Victimization among Adolescents in Europe: Baseline Results from the Lights4Violence Project. *International journal of environmental research and public health* 2021; **18**(4).

15. Wellman MM. Child sexual abuse and gender differences: Attitudes and prevalence. *Child Abuse and Neglect* 1993; **17**(4): 539-47.

16. Ackard DM, Neumark-Sztainer D. Multiple Sexual Victimizations among Adolescent Boys and Girls: Prevalence and Associations with Eating Behaviors and Psychological Health. *Journal of child sexual abuse* 2003; **12**(1): 17-37.

17. Ajduković M, Sušac N, Rajter M. Gender and age differences in prevalence and incidence of child sexual abuse in Croatia. *Croatian medical journal* 2013; **54**(5): 469-79.

18. Anwar Y, Sall M, Cislaghi B, et al. Assessing gender differences in emotional, physical, and sexual violence against adolescents living in the districts of Pikine and Kolda, Senegal. *Child Abuse and Neglect* 2020; **102**.

19. Bouvier P, Halpérin D, Rey HE, et al. Typology and correlates of sexual abuse in children and youth: Multivariate analyses in a prevalence study in Geneva. *Child Abuse and Neglect* 1999; **23**(8): 779-90.

20. Carrellas AZ. Sexual Victimization and Intellectual Disabilities among Adolescents Involved in Child Welfare; 2018.

21. Chandraratne NK, Fernando AD, Gunawardena N. Physical, sexual and emotional abuse during childhood: Experiences of a sample of Sri Lankan Young adults. *Child Abuse and Neglect* 2018; **81**: 214-24.

22. Chavez Ayala R, Rivera-Rivera L, Angeles-Llerenas A, Diaz-Ceron E, Allen-Leigh B, Lazcano Ponce E. Factors for sexual abuse during childhood and adolescence in students of Morelos, Mexico. *Revista de saude publica* 2009; **43**(3).

23. Emery CR, Wong PWC, Haden-Pawlowski V, et al. Neglect, online invasive exploitation, and childhood sexual abuse in Hong Kong: Breaking the links. *Child Abuse and Neglect* 2024; **147**.

24. Finkelhor D, Shattuck A, Turner HA, Hamby SL. The lifetime prevalence of child sexual abuse and sexual assault assessed in late adolescence. *Journal of Adolescent Health* 2014; **55**(3): 329-33.

25. Goessmann K, Ssenyonga J, Nkuba M, Hermenau K, Hecker T. Characterizing the prevalence and contributing factors of sexual violence: A representative cross-sectional study among school-going adolescents in two East African countries. *Child Abuse and Neglect* 2020; **109**.

26. Helweg-Larsen K, Larsen HB. The prevalence of unwanted and unlawful sexual experiences reported by Danish adolescents: Results from a national youth survey in 2002. *Acta Paediatrica, International Journal of Paediatrics* 2006; **95**(10): 1270-6.

27. Mansbach-Kleinfeld I, Ifrah A, Apter A, Farbstein I. Child sexual abuse as reported by Israeli adolescents: Social and health related correlates. *Child Abuse and Neglect* 2015; **40**: 68-80.

28. Mohler-Kuo M, Landolt MA, Maier T, Meidert U, Schoenbucher V, Schnyder U. Child Sexual Abuse Revisited: A Population-Based Cross-Sectional Study Among Swiss Adolescents. *JOURNAL OF ADOLESCENT HEALTH* 2014; **54**(3): 304-+.

29. Parent S, Lavoie F, Thibodeau MÈ, Hébert M, Blais M. Sexual Violence Experienced in the Sport Context by a Representative Sample of Quebec Adolescents. *Journal of interpersonal violence* 2016; **31**(16): 2666-86.

30. Wolitzky-Taylor KB, Ruggiero KJ, Danielson CK, et al. Prevalence and correlates of dating violence in a national sample of adolescents. *Journal of the American Academy of Child and Adolescent Psychiatry* 2008; **47**(7): 755-62.

31. Yen CF, Yang MS, Yang MJ, Su YC, Wang MH, Lan CM. Childhood physical and sexual abuse: Prevalence and correlates among adolescents living in rural Taiwan. *Child Abuse and Neglect* 2008; **32**(3): 429-38.

32. Bagley C. IS THE PREVALENCE OF CHILD SEXUAL ABUSE DECREASING - EVIDENCE FROM A RANDOM SAMPLE OF 750 YOUNG-ADULT WOMEN. *Psychological reports* 1990; **66**(3): 1037-8.

33. Cenat JM, Dalexis RD, Clormeus LA, et al. Lifetime and Child Sexual Violence, Risk Factors and Mental Health Correlates Among a Nationally Representative Sample of Adolescents and Young Adults in Haiti: A Public Health Emergency. *Journal of interpersonal violence* 2023; **38**(3-4): 2778-805.

34. Christoffersen MN, Armour C, Lasgaardc M, Andersenc TE, Elklitc A. The prevalence of four types of childhood maltreatment in denmark. *Clinical Practice and Epidemiology in Mental Health* 2013; **9**: 149-56.

35. Ernst C, Angst J, Földényi M. The zurich study - XVII. Sexual abuse in childhood. Frequency and relevance for adult morbidity data of a longitudinal epidemiological study. *European archives of psychiatry and clinical neuroscience* 1993; **242**(5): 293-300.

36. Aslam MV, Swedo E, Niolon PH, Peterson C, Bacon S, Florence C. Adverse Childhood Experiences Among U.S. Adults: National and State Estimates by Adversity Type, 2019–2020. *American journal of preventive medicine* 2024; **67**(1): 55-66.

37. Baker AW, Duncan SP. Child sexual abuse: A study of prevalence in Great Britain. *Child Abuse and Neglect* 1985; **9**(4): 457-67.

38. Langeland W, Hoogendoorn AW, Mager D, Smit JH, Draijer N. Childhood sexual abuse by representatives of the Roman Catholic Church: A prevalence estimate among the Dutch population. *Child Abuse and Neglect* 2015; **46**: 67-77.

39. LÓpez F, Hernández A. Child sexual abuse: Concept, prevalence and effects. *Infancia y Aprendizaje* 1995; **18**(71): 77-98.

40. Peltzer K, Pengpid S. Childhood physical and sexual abuse, and adult health risk behaviours among university students from 24 countries in Africa, the Americas and Asia. *Journal of Psychology in Africa* 2016; **26**(2): 149-55.

41. Perez-Fuentes G, Olfson M, Villegas L, Morcillo C, Wang S, Blanco C. Prevalence and correlates of child sexual abuse: a national study. *Comprehensive psychiatry* 2013; **54**(1): 16-27.

42. Speizer IS, Goodwin M, Whittle L, Clyde M, Rogers J. Dimensions of child sexual abuse before age 15 in three Central American countries: Honduras, El Salvador, and Guatemala. *Child Abuse and Neglect* 2008; **32**(4): 455-62.

43. Collings SJ. Childhood sexual abuse in a sample of South African university males: Prevalence and risk factors. *South African Journal of Psychology* 1991; **21**(3): 153-8.

44. Madu SN. The prevalence and patterns of childhood sexual abuse and victim-perpetrator relationship among a sample of university students. *South African Journal of Psychology* 2001; **31**(4): 32-7.

45. McCrann D, Lalor K, Katabaro JK. Childhood sexual abuse among university students in Tanzania. *Child Abuse and Neglect* 2006; **30**(12): 1343-51.

46. Meinck F, Cluver LD, Boyes ME, Loening-Voysey H. Physical, emotional and sexual adolescent abuse victimisation in South Africa: Prevalence, incidence, perpetrators and locations. *Journal of Epidemiology and Community Health* 2016; **70**(9): 910-6.

47. Mekuria A, Nigussie A, Abera M. Childhood sexual abuse experiences and its associated factors among adolescent female high school students in Arbaminch town, Gammo Goffa zone, Southern Ethiopia: a mixed method study. *BMC international health and human rights* 2015; **15**(1).

48. Rumble L, Mungate T, Chigiji H, et al. Childhood sexual violence in Zimbabwe: Evidence for the epidemic against girls. *Child Abuse and Neglect* 2015; **46**: 60-6.

49. Ward CL, Artz L, Leoschut L, Kassanjee R, Burton P. Sexual violence against children in South Africa: a nationally representative cross-sectional study of prevalence and correlates. *The Lancet Global Health* 2018; **6**(4): e460-e8.

50. Ashimi AO, Amole TG, Ugwa EA. Reported sexual violence among women and children seen at the gynecological emergency unit of a rural tertiary health facility, Northwest Nigeria. *ANNALS OF MEDICAL AND HEALTH SCIENCES RESEARCH* 2015; **5**(1): 26-9.

51. C MP, M CJ, Elias A, I OO, Awoere CT. Child sexual abuse among adolescents in southeast Nigeria: A concealed public health behavioral issue. *Pakistan journal of medical sciences* 2015; **31**(4): 827-32.

52. Chime OH, Orji CJ, Aneke TJ, Nwoke IN. Prevalence, pattern and predictors of child sexual abuse among senior secondary school students in enugu metropolis. *Malaysian Journal of Medical Sciences* 2021; **28**(4): 123-37.

53. Chinawa JM, Aronu AE, Chukwu BF, Obu HA. Prevalence and pattern of child abuse and associated factors in four secondary institutions in Enugu, Southeast Nigeria. *European journal of pediatrics* 2014; **173**(4): 451-6.

54. David N, Ezechi O, Wapmuk A, et al. Child sexual abuse and disclosure in south western nigeria: A community based study. *African Health Sciences* 2018; **18**(2): 199-208.

55. Owusu-Addo E, Owusu-Addo SB, Bennor DM, et al. Prevalence and determinants of sexual abuse among adolescent girls during the COVID-19 lockdown and school closures in Ghana: A mixed method study. *Child Abuse and Neglect* 2023; **135**.

56. Abdul Kadir NB, Desa A. Preliminary data on abuse during childhood among female university students. *Asia Pacific Journal of Social Work and Development* 2013; **23**(4): 299-314.

57. Chen J, Dunne MP, Han P. Child sexual abuse in China: A study of adolescents in four provinces. *Child Abuse and Neglect* 2004; **28**(11): 1171-86.

58. Chen J-q, Han P, Dunne MP. [Child sexual abuse: a study among 892 female students of a medical school]. *Zhonghua er ke za zhi = Chinese journal of pediatrics* 2004; **42**(1): 39-43.

59. Fanslow JL, Robinson EM, Crengle S, Perese L. Prevalence of child sexual abuse reported by a cross-sectional sample of New Zealand women. *Child Abuse and Neglect* 2007; **31**(9): 935-45.

60. Feng J-Y, Chang Y-T, Chang H-Y, Fetzer S, Wang J-D. Prevalence of different forms of child maltreatment among Taiwanese adolescents: A population-based study. *Child abuse & neglect* 2015; **42**: 10-9.

61. Fergusson DM, Lynskey MT, Horwood LJ. Childhood sexual abuse and psychiatric disorder in young adulthood .1. Prevalence of sexual abuse and factors associated with sexual abuse. *Journal of the American Academy of Child and Adolescent Psychiatry* 1996; **35**(10): 1355-64.

62. Goldman JDG, Padayachi UK. The prevalence and nature of child sexual abuse in Queensland, Australia. *Child Abuse and Neglect* 1997; **21**(5): 489-98.

63. Goldman RJ, Goldman JDG. The prevalence and nature of child sexual abuse in australia. *Australian Journal of Sex, Marriage and Family* 1988; **9**(2): 94-106.

64. Hamelin C, Salomon C, Cyr D, Gueguen A, Lert F. Childhood sexual abuse and adult sexual health among indigenous Kanak women and non-Kanak women of New Caledonia. *Child Abuse and Neglect* 2010; **34**(9): 677-88.

65. Kadir NByA, Desa A. Preliminary data on abuse during childhood among female university students. *ASIA PACIFIC JOURNAL OF SOCIAL WORK AND DEVELOPMENT* 2013; **23**(4): 299-314.

66. Kim HS, Kim HS. Incestuous experience among Korean adolescents: Prevalence, family problems, perceived family dynamics, and psychological characteristics. *Public Health Nursing* 2005; **22**(6): 472-82.

67. Ko Ling C, Yan E, Brownridge DA, Tiwari A, Fong DY. Childhood sexual abuse associated with dating partner violence and suicidal ideation in a representative household sample in Hong Kong. *Journal of interpersonal violence* 2011; **26**(9): 1763-84.

68. Li N, Zabin LS, Ahmed S. The childhood sexual abuse among youth in three asian cities: Taipei, shanghai, and hanoi. *Asia-Pacific journal of public health* 2015; **27**(2): NP1566-NP77.

69. Luo Y, Parish WL, Laumann EO. A population-based study of childhood sexual contact in China: prevalence and long-term consequences. *Child abuse & neglect* 2008; **32**(7): 721-31.

70. Mathews B, Finkelhor D, Pacella R, et al. Child sexual abuse by different classes and types of perpetrator: Prevalence and trends from an Australian national survey. *Child abuse & neglect* 2024; **147**.

71. Moran P, Coffey C, Chanen A, Mann A, Carlin JB, Patton GC. Childhood sexual abuse and abnormal personality: A population-based study. *Psychological medicine* 2011; **41**(6): 1311-8.

72. Tang CSK. Childhood experience of sexual abuse among Hong Kong Chinese college students. *Child Abuse and Neglect* 2002; **26**(1): 23-37.

73. Tang K, Qu X, Li C, Tan S. Childhood sexual abuse, risky sexual behaviors and adverse reproductive health outcomes among Chinese college students. *Child abuse & neglect* 2018; **84**: 123-30.

74. Telfar S, McLeod GFH, Dhakal B, et al. Child abuse and neglect and mental health outcomes in adulthood by ethnicity: Findings from a 40-year longitudinal study in New Zealand/Aotearoa. *Child Abuse and Neglect* 2023; **145**.

75. Thang Nguyen H, Anh Le V, Peltzer K, Pengpid S, Low WY, Win HH. Childhood Emotional, Physical, and Sexual Abuse and Associations With Mental Health and Health-Risk Behaviors Among University Students in the Association of Southeast Asian Nations (ASEAN). *CHILD STUDIES IN ASIA-PACIFIC CONTEXT* 2017; **7**(1): 15-26.

76. Akyuz G, Sar V, Kugu N, Doğan O. Reported childhood trauma, attempted suicide and self-mutilative behavior among women in the general population. *European Psychiatry* 2005; **20**(3): 268-73.

77. Arnarsson ÁM, Gísladóttir KH, Jónsson SH. The prevalence of sexual abuse and sexual assault against icelandic adolescents. *Laeknabladid* 2016; **102**(6): 289-95.

78. Baviskar S, Christensen E. Childhood sexual abuse of women in Greenland and its developmental correlates among their children. *International journal of circumpolar health* 2011; **70**(1): 29-36.

79. Bebbington PE, Jonas S, Brugha T, et al. Child sexual abuse reported by an English national sample: Characteristics and demography. *Social psychiatry and psychiatric epidemiology* 2011; **46**(3): 255-62.

80. Bellis MA, Hughes K, Leckenby N, Perkins C, Lowey H. National household survey of adverse childhood experiences and their relationship with resilience to health-harming behaviors in England. *BMC medicine* 2014; **12**(1).

81. Broekhof R, Nordahl HM, Bjørnelv S, Selvik SG. Prevalence of adverse childhood experiences and their co-occurrence in a large population of adolescents: a Young HUNT 3 study. *Social psychiatry and psychiatric epidemiology* 2022; **57**(12): 2359-66.

82. Castelli B, Festa F, Di Sanzo MA, Guala A, Pellai A. Prevalence of child sexual abuse: a comparison among 4 Italian epidemiological studies. *La Pediatria medica e chirurgica : Medical and surgical pediatrics* 2015; **37**(2): pmc.2015.114.

83. Denholm R, Power C, Thomas C, Li L. Child Maltreatment and Household Dysfunction in a British Birth Cohort. *CHILD ABUSE REVIEW* 2013; **22**(5): 340-53.

84. Dias A, Sales L, Hessen DJ, Kleber RJ. Child maltreatment and psychological symptoms in a Portuguese adult community sample: the harmful effects of emotional abuse. *European child & adolescent psychiatry* 2015; **24**(7): 767-78.

85. Euser S, Alink LRA, Tharner A, van Ijzendoorn MH, Bakermans-Kranenburg MJ. The Prevalence of Child Sexual Abuse in Out-of-Home Care: A Comparison Between Abuse in Residential and in Foster Care. *Child maltreatment* 2013; **18**(4): 221-31.

86. Fernández-García O, Gil-Llario MD, Ballester-Arnal R. Sexual Victimization of Adolescents in Residential Care: Self-Reported and Other-Reported Prevalence. *Journal of sex research* 2024; **61**(3): 389-98.

87. Ferragut M, Ortiz-Tallo M, Blanca MJ. Prevalence of Child Sexual Abuse in Spain: A Representative Sample Study. *Journal of interpersonal violence* 2022; **37**(21-22): NP19358-NP77.

88. Gerke J, Rassenhofer M, Witt A, Sachser C, Fegert JM. Female-Perpetrated Child Sexual Abuse: Prevalence Rates in Germany. *Journal of child sexual abuse* 2020; **29**(3): 263-77.

89. Ireland CA, Alderson K, Ireland JL. Sexual Exploitation in Children: Nature, Prevalence, and Distinguishing Characteristics Reported in Young Adulthood. *Journal of Aggression, Maltreatment and Trauma* 2015; **24**(6): 603-22.

90. Junco M, Ferragut M, Blanca MJ. Prevalence of Child Contact Sexual Abuse in the Spanish Region of Andalusia. *Journal of child sexual abuse* 2022; **31**(8): 892-910.

91. Kuhar M, Zavirsek D. Prevalence and Characteristics of Child Sexual Abuse in Slovenia. *REVIJA ZA KRIMINALISTIKO IN KRIMINOLOGIJO* 2023; **74**(1): 51-62.

92. Lopez F, Carpintero E, Hernandez A, Martin MJ, Fuertes A. PREVALENCE AND CONSEQUENCES OF SEXUAL ABUSE IN CHILDREN IN SPAIN. *Child abuse & neglect* 1995; **19**(9): 1039-50.

93. López S, Faro C, Lopetegui L, et al. Child and Adolescent Sexual Abuse in Women Seeking Help for Sexual and Reproductive Mental Health Problems: Prevalence, Characteristics, and Disclosure. *Journal of child sexual abuse* 2017; **26**(3): 246-69.

94. Marcos V, Treskow L, Kuester R, Seijo D. SEXUAL VIOLENCE VICTIMIZATION: PREVALENCE IN ADOLESCENTS AND DISPOSITION TO FORGIVE. *ACCION PSICOLOGICA* 2023; **20**(2): 113-24.

95. Mateus MISMZ. Prevalence of Sexual Abuse in Portuguese Children; 2012.

96. Okur P, Van Der Knaap LM, Bogaerts S. Research on prevalence and media coverage of child sexual abuse - Prevalence and nature of child sexual abuse in the Netherlands: Ethnic differences? *Journal of child sexual abuse* 2015; **24**(1): 1-15.

97. Pedersen W, Bakken A, Stefansen K, von Soest T. Sexual Victimization in the Digital Age: A Population-Based Study of Physical and Image-Based Sexual Abuse Among Adolescents. *Archives of sexual behavior* 2023; **52**(1): 399-410.

98. Veldwijk J, Proper KI, Hoeven-Mulder HB, Bemelmans WJ. The prevalence of physical, sexual and mental abuse among adolescents and the association with BMI status. *BMC public health* 2012; **12**: 840.

99. Vives-Cases C, Perez-Martinez V, Davo-Blanes M, et al. Dating violence and associated factors among male and female adolescents in Spain. *PloS one* 2021; **16**(11).

100. Barthauer LM, Leventhal JM. Prevalence and effects of child sexual abuse in a poor, rural community in El Salvador: A retrospective study of women after 12 years of civil war. *Child Abuse and Neglect* 1999; **23**(11): 1117-26.

101. Bassani DG, Palazzo LS, Beria JU, et al. Child sexual abuse in southern Brazil and associated factors: a population-based study. *BMC public health* 2009; **9**.

102. Diehl A, Molina de Souza R, Madruga CS, Laranjeira R, Wagstaff C, Pillon SC. Rape, Child Sexual Abuse, and Mental Health in a Brazilian National Sample. *Journal of interpersonal violence* 2022; **37**(1-2): NP944-NP67.

103. Feitosa SO, Noll M, Mendonca CR, Silveira EA, Esposito Sorpreso IC, Silva Noll PRE. Prevalence of sexual abuse and its association with health-risk behaviors among Brazilian adolescents: A populational study. *Child abuse & neglect* 2021; **122**.

104. van der Kooij IW, Bipat S, Nieuwendam J, Lindauer RJL, Graafsma TLG. The Prevalence of Sexual Abuse in Adolescence in Suriname. *Journal of child sexual abuse* 2019; **28**(4): 435-51.

105. Afifi ZEM, El-Lawindi MI, Ahmed SA, Basily WW. Adolescent abuse in a community sample in Beni Suef, Egypt: Prevalence and risk factors. *Eastern Mediterranean Health Journal* 2003; **9**(5-6): 1003-18.

106. Al-Eissa MA, AlBuhairan FS, Qayad M, Saleheen H, Runyan D, Almuneef M. Determining child maltreatment incidence in Saudi Arabia using the ICAST-CH: A pilot study. *Child Abuse and Neglect* 2015; **42**: 174-82.

107. Al-Fayez GA, Ohaeri JU, Gado OM. Prevalence of physical, psychological, and sexual abuse among a nationwide sample of Arab high school students: association with family characteristics, anxiety, depression, self-esteem, and quality of life. *Social psychiatry and psychiatric epidemiology* 2012; **47**(1): 53-66.

108. Al-Zboon E, Ahmad J, Al-Dababneh K. Prevalence and types of childhood abuse among special education students attending Jordanian Universities. *International Journal of Adolescence and Youth* 2016; **21**(4): 476-85.

109. Aldharman SS, Alrasheed LS, Alotaibi WS, et al. Determining the Prevalence of Child Maltreatment Among Young Adults in Saudi Arabia Using ISPCAN Child Abuse Screening Tool. *CUREUS JOURNAL OF MEDICAL SCIENCE* 2023; **15**(5).

110. Almazeedi H, Alkandari S, Alrazzuqi H, Ohaeri J, Alfayez G. Prevalence of child abuse and its association with depression among first year students of Kuwait University: a cross-sectional study. *Eastern Mediterranean health journal = La revue de sante de la Mediterranee orientale = al-Majallah al-sihhiyah li-sharq al-mutawassit* 2020; **26**(8): 948-56.

111. Danaeifar M, Arshi M, Moghanibashi-Mansourieh A. Child sexual abuse in Iran: a systematic review of the prevalence, risk factors, consequences, interventions and laws. *Journal of injury & violence research* 2022; **14**(3).

112. Ibrahim NK, Jalali EA, Al-Ahmadi JR, Al-Bar AA. Prevalence, risk factors and outcome of childhood abuse reported by female university students in jeddah. *The Journal of the Egyptian Public Health Association* 2008; **83**(5-6): 329-51.

113. Jumaian A. Prevalence and long-term impact of Child Sexual Abuse among a sample of male college students in Jordan. *Eastern Mediterranean Health Journal* 2001; **7**(3): 435-40.

114. Schein M, Biderman A, Baras M, et al. The prevalence of a history of child sexual abuse among adults visiting family practitioners in Israel. *Child Abuse and Neglect* 2000; **24**(5): 667-75.

115. Usta J, Farver J. Child sexual abuse in Lebanon during war and peace. *Child: care, health and development* 2010; **36**(3): 361-8.

116. Vakilian K, Mousavi SA, Keramat A. Child sexual abuse based on the crosswise model: A cross-sectional study on 18–24-year-old iranian students. *Family Medicine and Primary Care Review* 2019; **21**(3): 249-52.

117. Vega E, Mashiach RT. Awareness, Incidence and Psychological Wellbeing of Childhood Sexual Abuse as Reported by Ultra-Orthodox Mothers. *Journal of child sexual abuse* 2023; **32**(5): 554-74.

118. Ackard DM, Neumark-Sztainer D. Date violence and date rape among adolescents: Associations with disordered eating behaviors and psychological health. *Child Abuse and Neglect* 2002; **26**(5): 455-73.

119. Amodeo M, Griffin ML, Fassler IR, Clay CM, Ellis MA. Childhood sexual abuse among black women and white women from two-parent families. *Child maltreatment* 2006; **11**(3): 237-46.

120. Barnert ES, Bath E, Heard-Garris N, et al. Commercial Sexual Exploitation During Adolescence: A US-Based National Study of Adolescent to Adult Health. *Public health reports (Washington, DC : 1974)* 2022; **137**(1_suppl): 53s-62s.

121. Briere J, Runtz M, Rodd K. Child and Adolescent Exposure to Sexual Harassment: Relationship to Gender, Contact Sexual Abuse, and Adult Psychological Symptoms. *Journal of interpersonal violence* 2024; **39**(13-14): 2981-96.

122. Burcham B, Leachman M, Luftman V. Relationship and Dating Violence in School-Aged Adolescents. School Violence and Primary Prevention, Second Edition; 2023: 261-93.

123. Chen CT, Yang NP, Chou P. Child maltreatment in Taiwan for 2004-2013: A shift in age group and forms of maltreatment. *Child Abuse and Neglect* 2016; **52**: 169-76.

124. Chiu GR, Lutfey KE, Litman HJ, Link CL, Hall SA, McKinlay JB. Prevalence and overlap of childhood and adult physical, sexual, and emotional abuse: A descriptive analysis of results from the boston area community health (BACH) survey. *Violence and victims* 2013; **28**(3): 381-402.

125. Daigneault I, Hébert M, McDuff P. Men's and women's childhood sexual abuse and victimization in adult partner relationships: A study of risk factors. *Child Abuse and Neglect* 2009; **33**(9): 638-47.

126. Dawson CJZ. Gender differences in risk factors and moderators of the relationships between known childhood risk factors and sexually coercive behavior in a young adult community sample; 2014.

127. Finkelhor D, Turner H, Colburn D. The prevalence of child sexual abuse with online sexual abuse added. *Child Abuse and Neglect* 2024; **149**.

128. Foshee VA. Gender differences in adolescent dating abuse prevalence, types and injuries. *Health education research* 1996; **11**(3): 275-86.

129. Freedner N, Freed LH, Yang YW, Austin SB. Dating violence among gay, lesbian, and bisexual adolescents: Results from a community survey. *Journal of Adolescent Health* 2002; **31**(6): 469-74.

130. Hébert M, Amédée LM, Blais M, Gauthier-Duchesne A. Child Sexual Abuse among a Representative Sample of Quebec High School Students: Prevalence and Association with Mental Health Problems and Health-Risk Behaviors. *Canadian Journal of Psychiatry* 2019; **64**(12): 846-54.

131. Hemmings RZ. Teen Dating Violence: Co-Occurrence with Bullying among African American Teens in South Florida; 2016.

132. Hilton NZ, Jennings KT, Drugge J, Stephens J. Childhood Sexual Abuse Among Clinicians Working With Sex Offenders. *Journal of interpersonal violence* 1995; **10**(4): 525-32.

133. Kenny MC, McEachern AG. Prevalence and characteristics of childhood sexual abuse in multiethnic female college students. *Journal of child sexual abuse* 2000; **9**(2): 57-70.

134. Kogan SM. Disclosing unwanted sexual experiences: Results from a national sample of adolescent women. *Child Abuse and Neglect* 2004; **28**(2): 147-65.

135. Nussbaum BRZ. Sexual offending and victimization: Prevalence and reactions of adolescent and adult sex offenders and college students; 1990.

136. Pineda-Lucatero AG, Trujillo-Hernandez B, Millan-Guerrero RO, Vasquez C. Prevalence of childhood sexual abuse among Mexican adolescents. *CHILD CARE HEALTH AND DEVELOPMENT* 2009; **35**(2): 184-9.

137. Robertson HA, Chaudhary Nagaraj N, Vyas AN. Family Violence and Child Sexual Abuse Among South Asians in the US. *Journal of immigrant and minority health* 2016; **18**(4): 921-7.

138. Saewyc EM, Pettingell S, Magee LL. The prevalence of sexual abuse among adolescents in school. *The Journal of school nursing : the official publication of the National Association of School Nurses* 2003; **19**(5): 266-72.

139. Shields M, Tonmyr L, Hovdestad WE. The Decline of Child Sexual Abuse in Canada: Evidence From the 2014 General Social Survey. *Canadian Journal of Psychiatry* 2019; **64**(9): 638-46.

140. Siegel JM, Sorenson SB, Golding JM, Burnam MA, Stein JA. The prevalence of childhood sexual assault: The los angeles epidemiologic catchment area project. *American journal of epidemiology* 1987; **126**(6): 1141-53.

141. Silverman JG, Raj A, Mucci LA, Hathaway JE. Dating violence against adolescent girls and associated substance use, unhealthy weight control, sexual risk behavior, pregnancy, and suicidality. *Jama* 2001; **286**(5): 572-9.

142. Symons PY, Groër MW, Kepler-Youngblood P, Slater V. Prevalence and Predictors of Adolescent Dating Violence. *Journal of Child and Adolescent Psychiatric Nursing* 1994; **7**(3): 14-23.

143. Taylor S, Xia Y. Dating Violence Among Rural Adolescents: Perpetration and Victimization by Gender. *Journal of interpersonal violence* 2022; **37**(9-10): Np7729-np50.

144. Vogeltanz ND, Wilsnack SC, Harris TR, Wilsnack RW, Wonderlich SA, Kristjanson AF. Prevalence and risk factors for childhood sexual abuse in women: National survey findings. *Child Abuse and Neglect* 1999; **23**(6): 579-92.

145. Wyatt GE. The sexual abuse of Afro-American and White-American women in childhood. *Child Abuse and Neglect* 1985; **9**(4): 507-19.

146. Wyatt GE, Burns Loeb T, Solis B, Vargas Carmona J, Romero G. The prevalence and circumstances of child sexual abuse: Changes across a decade. *Child Abuse and Neglect* 1999; **23**(1): 45-60.

147. Young MSZ. Prevalence and sequelae of childhood sexual abuse among male undergraduates; 2004.

148. Daral S, Khokhar A, Pradhan S. Prevalence and determinants of child maltreatment among school-going adolescent girls in a semi-urban area of Delhi, India. *Journal of tropical pediatrics* 2016; **62**(3): 227-40.

149. Delanthabettu H, Pandiyan K, Kaveri SK, Gunari RV, Vijayakumar SN. A STUDY ON CHILD SEXUAL ABUSE IN SCHOOL GOING CHILDREN. *JOURNAL OF EVOLUTION OF MEDICAL AND DENTAL SCIENCES-JEMDS* 2017; **6**(67): 4798-801.

150. Jangam K, Muralidharan K, Tansa KA, Aravind Raj E, Bhowmick P. Incidence of childhood abuse among women with psychiatric disorders compared with healthy women: Data from a tertiary care centre in India. *Child Abuse and Neglect* 2015; **50**: 67-75.

151. Krishnakumar P, Satheesan K, Geeta MG, Sureshkumar K. Prevalence and spectrum of sexual abuse among adolescents in Kerala, South India. *Indian journal of pediatrics* 2014; **81**(8): 770-4.

152. Perera B, Ostbye T. Prevalence and correlates of sexual abuse reported by late adolescent school children in Sri Lanka. *International journal of adolescent medicine and health* 2009; **21**(2): 203-11.

153. Shrestha S, Baskota S, Karki U, et al. Child sexual abuse among school children of a municipality: A descriptive cross-sectional study. *Journal of the Nepal Medical Association* 2021; **59**(239): 672-7.

154. Buttar A, Clements-Nolle K, Haas J, Reese F. Dating Violence, Psychological Distress, and Attempted Suicide Among Female Adolescents in the Juvenile Justice System. *Journal of Correctional Health Care* 2013; **19**(2): 101-12.

155. Clark VA, Duwe G. Sex Differences in the Effects of Adverse Childhood Experiences on Institutional Misconduct among Adults in Prison. *Journal of interpersonal violence* 2024: 8862605241246799.

156. Coleman D, Stewart LM. Prevalence and impact of childhood maltreatment in incarcerated youth. *American Journal of Orthopsychiatry* 2010; **80**(3): 343-9.

157. Johnson RJ, Ross MW, Taylor WC, Williams ML, Carvajal RI, Peters RJ. Prevalence of childhood sexual abuse among incarcerated males in county jail. *Child Abuse and Neglect* 2006; **30**(1): 75-86.

158. Pagare D, Meena GS, Jiloha RC, Singh MM. Sexual abuse of street children brought to an observation home. *Indian pediatrics* 2005; **42**(2): 134-1139.

159. Abbes W, Kerkeni A, Emna E. Prevalence of Childhood Sexual Abuse Among Tunisian Psychiatric Outpatients and its Associated Factors. *Iranian Journal of Psychiatry and Clinical Psychology* 2024; **29**(4): 514-31.

160. Agyapong VIO, Juhás M, Ritchie A, Ogunsina O, Ambrosano L, Corbett S. Prevalence Rate and Demographic and Clinical Correlates of Child Sexual Abuse Among New Psychiatric Outpatients in a City in Northern Alberta. *Journal of child sexual abuse* 2017; **26**(4): 442-52.

161. Cloitre M, Tardiff K, Marzuk PM, Leon AC, Portera L. Childhood abuse and subsequent sexual assault among female inpatients. *Journal of traumatic stress* 1996; **9**(3): 473-82.

162. Green SM, Russo MF, Navratil JL, Loeber R. Sexual and physical abuse among adolescent girls with disruptive behavior problems. *Journal of Child and Family Studies* 1999; **8**(2): 151-68.

163. Koola MM, Qualls C, Kelly DL, et al. Prevalence of childhood physical and sexual abuse in veterans with psychiatric diagnoses. *The Journal of nervous and mental disease* 2013; **201**(4): 348-52.

164. Morris PA, Bihan SM. The Prevalence of Children with a History of Sexual Abuse Hospitalized in the Psychiatric Setting. *Journal of Child and Adolescent Psychiatric Nursing* 1991; **4**(2): 49-54.

165. Romero S, Birmaher B, Axelson D, et al. Prevalence and correlates of physical and sexual abuse in children and adolescents with bipolar disorder. *Journal of affective disorders* 2009; **112**(1-3): 144-50.

166. Ross CA, Keyes BB, Xiao Z, et al. Childhood physical and sexual abuse in China. *Journal of child sexual abuse* 2005; **14**(4): 115-26.

167. Threlkeld ME, Thyer BA. Sexual and physical abuse histories among child and adolescent psychiatric outpatients. *Journal of traumatic stress* 1992; **5**(3): 491-6.

168. Wright B, West D, Worrall A, Tagg G. Prevalence of confirmed child abuse and the use of resources in child psychiatric out-patients. *Psychiatric Bulletin* 1996; **20**(4): 207-9.

169. Wurr CJ, Partridge IM. The prevalence of a history of childhood sexual abuse in an acute adult inpatient population. *Child Abuse and Neglect* 1996; **20**(9): 867-72.

170. Arreola SG, Neilands TB, Pollack LM, Paul JP, Catania JA. Higher prevalence of childhood sexual abuse among Latino men who have sex with men than non-Latino men who have sex with men: data from the Urban Men's Health Study. *Child abuse & neglect* 2005; **29**(3): 285-90.

171. El Khoury C, Mutchler MG, Ghanem CA, et al. Sexual Violence in Childhood and Post-Childhood: The Experiences of Young Men Who Have Sex With Men in Beirut. *Journal of interpersonal violence* 2021; **36**(19-20): NP11198-NP217.

172. Ferreira DG, Veras MA, Saggese GSR, et al. Prevalence, Characteristics, and Factors Associated With Sexual Violence in Adulthood Among Brazilian MSM. *American journal of men's health* 2022; **16**(6): 15579883221142173.

173. Levine EC, Martinez O, Mattera B, et al. Child Sexual Abuse and Adult Mental Health, Sexual Risk Behaviors, and Drinking Patterns Among Latino Men Who Have Sex With Men. *Journal of child sexual abuse* 2018; **27**(3): 237-53.

174. Sauceda JA, Wiebe JS, Simoni JM. Childhood sexual abuse and depression in Latino men who have sex with men: Does resilience protect against nonadherence to antiretroviral therapy? *Journal of health psychology* 2016; **21**(6): 1096-106.

175. Tomori C, McFall AM, Srikrishnan AK, et al. The prevalence and impact of childhood sexual abuse on HIV-risk behaviors among men who have sex with men (MSM) in India. *BMC public health* 2016; **16**: 784.

176. Xu W, Zheng L, Zheng Y. Prevalence of non-contact and contact childhood sexual abuse: An Internet-based sample of men who have sex with men in China. *PloS one* 2017; **12**(4): e0175444.

177. Chimdessa A, Cheire A. Sexual and physical abuse and its determinants among street children in Addis Ababa, Ethiopia 2016. *BMC pediatrics* 2018; **18**(1): 304.

178. Noell J, Rohde P, Seeley J, Ochs L. Childhood sexual abuse, adolescent sexual coercion and sexually transmitted infection acquisition among homeless female adolescents. *Child Abuse and Neglect* 2001; **25**(1): 137-48.

179. Rew L, Taylor-Seehafer M, Fitzgerald ML. Sexual abuse, alcohol and other drug use, and suicidal behaviors in homeless adolescents. *Issues in comprehensive pediatric nursing* 2001; **24**(4): 225-40.

180. Zhao Q, Zhao J, Li X, et al. Childhood sexual abuse and its relationship with psychosocial outcomes among children affected by HIV in rural China. *Journal of the Association of Nurses in AIDS Care* 2011; **22**(3): 202-14.

181. Helmus LM, Kyne A. Prevalence, Correlates, and Sequelae of Child Sexual Abuse (CSA) among Indigenous Canadians: Intersections of Ethnicity, Gender, and Socioeconomic Status. *International journal of environmental research and public health* 2023; **20**(9).

182. Robin RW, Chester B, Rasmussen JK, Jaranson JM, Goldman D. Prevalence, characteristics, and impact of childhood sexual abuse in a southwestern American Indian tribe. *Child Abuse and Neglect* 1997; **21**(8): 769-87.

183. Balogh R, Bretherton K, Whibley S, et al. Sexual abuse in children and adolescents with intellectual disability. *Journal of Intellectual Disability Research* 2001; **45**(3): 194-201.

184. Hershkowitz I, Horowitz D, Lamb ME. Trends in children's disclosure of abuse in Israel: A national study. *Child Abuse and Neglect* 2005; **29**(11): 1203-14.

185. Kvam MH. Sexual abuse of deaf children. A retrospective analysis of the prevalence and characteristics of childhood sexual abuse among deaf adults in Norway. *Child Abuse and Neglect* 2004; **28**(3): 241-51.

186. Kvam MH. Experiences of childhood sexual abuse among visually impaired adults in Norway: Prevalence and characteristics. *Journal of Visual Impairment and Blindness* 2005; **99**(1): 5-14.

187. Abera L, Aliye A, Tadesse K, Guta A. Magnitude of child sexual abuse and its associated factors among high school female students in Dire Dawa, Eastern Ethiopia: a cross-sectional study. *Reproductive health* 2021; **18**(1).

188. Andersson N, Paredes-Solís S, Milne D, et al. Prevalence and risk factors for forced or coerced sex among school-going youth: national cross-sectional studies in 10 southern African countries in 2003 and 2007. *BMJ open* 2012; **2**(2): e000754.

189. Kunnuji MON, Esiet A. Prevalence and correlates of sexual abuse among female out-of-school adolescents in Iwaya community, Lagos State, Nigeria. *African journal of reproductive health* 2015; **19**(1): 82-90.

190. Manyike PC, Chinawa JM, Aniwada E, Udechukwu NP, Odutola OI, Chinawa TA. Child sexual abuse among adolescents in southeast Nigeria: A concealed public health behavioral issue. *Pakistan journal of medical sciences* 2015; **31**(4): 827-32.

191. Worku D, Gebremariam A, Jayalakshmi S. Child sexual abuse and its outcomes among high school students in southwest Ethiopia. *Tropical Doctor* 2006; **36**(3): 137-40.

192. Abbas SS, Jabeen T. Prevalence of Child Abuse Among the University Students: A Retrospective Cross-Sectional Study in University of the Punjab, Pakistan. *International Quarterly of Community Health Education* 2020; **40**(2): 125-34.

193. Aguilar A, Salcedo M. Characteristics of sexual violence in adolescents from 10 to 19 years of age, Cali 2001-2003. *Colombia Medica* 2008; **39**(4): 356-63.

194. Alemayehu B, Tafesse N, Chanyalew E. Magnitude of Child Sexual Abuse and Its Associated Factors Among Children Treated in Public Hospitals of Addis Ababa Ethiopia. *Adolescent health, medicine and therapeutics* 2022; **13**: 67-76.

195. Bhowate S, Asawa S. Child sexual abuse - An area of emerging concern. *Indian Journal of Forensic Medicine and Toxicology* 2015; **9**(2): 40-5.

196. Chacko AZ, Paul JSG, Vishwanath R, et al. A study on child sexual abuse reported by urban indian college students. *Journal of family medicine and primary care* 2022; **11**(9): 5072-6.

197. Gwirayi P. The prevalence of child sexual abuse among secondary school pupils in Gweru, Zimbabwe. *Journal of Sexual Aggression* 2013; **19**(3): 253-63.

198. Senn TE, Carey MP, Vanable PA, Coury-Doniger P, Urban MA. Childhood sexual abuse and sexual risk behavior among men and women attending a sexually transmitted disease clinic. *Journal of consulting and clinical psychology* 2006; **74**(4): 720-31.

199. Steiner JJ, Johnson L, Postmus JL, Davis R. Sexual Violence of Liberian School Age Students: An Investigation of Perpetration, Gender, and Forms of Abuse. *Journal of child sexual abuse* 2021; **30**(1): 21-40.

200. Takele M, Haye TB, Kitaw LD, Uqubay N, Gebremedhin KB. Overview of child sexual and substance abuse among children in Ethiopia. *Journal of family medicine and primary care* 2020; **9**(12): 6140-6.

201. Anderson J, Martin J, Mullen P, Romans S, Herbison P. Prevalence of Childhood Sexual Abuse Experiences in a Community Sample of Women. *Journal of the American Academy of Child and Adolescent Psychiatry* 1993; **32**(5): 911-9.

202. Biluala F, Kayembe HC, Batumbo D, et al. Magnitude and factors associated with sexual re-victimization among adolescent girls and young women in Kinshasa, Democratic Republic of the Congo: a retrospective multicenter study. *Reproductive health* 2023; **20**(1).

203. Kercher GA, McShane M. The prevalence of child sexual abuse victimization in an adult sample of Texas residents. *Child abuse & neglect* 1984; **8**(4): 495-501.

204. MacMillan HL, Fleming JE, Trocmé N, et al. Prevalence of child physical and sexual abuse in the community: Results from the Ontario Health Supplement. *Jama* 1997; **278**(2): 131-5.

205. Cappelleri JC, Eckenrode J, Powers JL. The epidemiology of child abuse: Findings from the Second National Incidence and Prevalence Study of Child Abuse and Neglect. *American journal of public health* 1993; **83**(11): 1622-4.

206. Hawke JM, Jainchill N, Leon GD. The prevalence of sexual abuse and its impact on the onset of drug use among adolescents in therapeutic community drug treatment. *Journal of Child and Adolescent Substance Abuse* 2000; **9**(3): 35-49.

207. Agbaje OS, Nnaji CP, Nwagu EN, et al. Adverse childhood experiences and psychological distress among higher education students in Southeast Nigeria: an institutional-based cross-sectional study. *Archives of Public Health* 2021; **79**(1).

208. Craven S, Brown S, Gilchrist E. Sexual grooming of children: Review of literature and theoretical considerations. *Journal of Sexual Aggression* 2006; **12**(3): 287-99.

209. Doyle RLZ. Childhood abuse and adverse experience in adolescents who harm others. 2014.

210. Falbo G, Caminha F, Aguiar F, et al. Incidence of child and adolescent abuse among incarcerated females in the Northeast of Brazil. *Journal of tropical pediatrics* 2004; **50**(5): 292-6.

211. Mrazek PJ, Lynch MA, Bentovim A. Sexual abuse of children in the United Kingdom. *Child Abuse and Neglect* 1983; **7**(2): 147-53.

212. O'Reilly G, Carr A. Child sexual abuse in Ireland: A synthesis of two surveys. *Irish Journal of Psychology* 1999; **20**(1): 1-14.

213. Pereda N, Abad J, Guilera G. Lifetime Prevalence and Characteristics of Child Sexual Victimization in a Community Sample of Spanish Adolescents. *Journal of child sexual abuse* 2016; **25**(2): 142-58.

214. Thomsen L, Thompson C, Ogilvie J, et al. Child Sexual Abuse Victimization Amongst Detained Adolescents and Incarcerated Young Adults: Findings from an Australian Population-Based Birth Cohort Study. *Journal of child sexual abuse* 2024; **33**(4): 465-84.

215. Zabaneh NFZ. The prevalence of sexual abuse and substance abuse among dual-diagnosed adolescents; 2003.

216. Ayaya S, DeLong A, Embleton L, et al. Prevalence, incidence and chronicity of child abuse among orphaned, separated, and street-connected children and adolescents in western Kenya: What is the impact of care environment? *Child Abuse and Neglect* 2023; **139**.

217. Debowska A, Boduszek D. Child abuse and neglect profiles and their psychosocial consequences in a large sample of incarcerated males. *Child Abuse and Neglect* 2017; **65**: 266-77.

218. de Almeida Ramos KA, Russo Rafael RdM, Garcia Penna LH, Depret DG, Ribeiro LV, Carinhanha JI. Sheltered adolescents' background of exposure to violence and distressful experiences. *REVISTA BRASILEIRA DE ENFERMAGEM* 2020; **73**(4).

219. Bhullar DS, Mittal P, Sidhu BS, Dipti B. Sexual abuse - Councelling and prevalence in female school children in Patiala. *Journal of Punjab Academy of Forensic Medicine and Toxicology* 2017; **17**(1): 27-31.

220. Carlstedt A, Forsman A, Soderstrom H. Sexual child abuse in a defined Swedish area 1993-97: A population-based survey. *Archives of sexual behavior* 2001; **30**(5): 483-93.

221. Csorba R, Aranyosi J, Borsos A, Balla L, Major T, Póka R. Characteristics of female child sexual abuse in Hungary between 1986 and 2001: A longitudinal, prospective study. *European Journal of Obstetrics and Gynecology and Reproductive Biology* 2005; **120**(2): 217-21.

222. Delziovo CR, Bolsoni CC, Nazário NO, Coelho EBS. Characteristics of sexual violence against adolescent and adult women reported by the public health services in Santa Catarina State, Brazil. *Cadernos de saude publica* 2017; **33**(6).

223. Dunne MP, Purdie DM, Cook MD, Boyle FM, Najman JM. Is child sexual abuse declining? Evidence from a population-based survey of men and women in Australia. *Child Abuse and Neglect* 2003; **27**(2): 141-52.

224. Farmer A. Sexual Exploitation, Abuse, and Trafficking of School-Aged Children. School Violence and Primary Prevention, Second Edition; 2023: 231-59.

225. Catarina Almeida T, Barreiros I. Online grooming among Portuguese adolescents and the COVID-19 lockdown: Relationship with other types of victimization. *Children and Youth Services Review* 2024; **156**.

226. Munroe C, Shumway M. Female-Perpetrated Sexual Violence: A Survey of Survivors of Female-Perpetrated Childhood Sexual Abuse and Adult Sexual Assault. *Journal of interpersonal violence* 2022; **37**(9-10): NP6655-NP75.

227. Alonso-Ruido P, Estévez I, Regueiro B, Varela-Portela C. Victims of Child Grooming: An Evaluation in University Students. *Societies* 2024; **14**(1).

228. Csorba R, Tsikouras P, Lampé R, Póka R. The sexual abuse of female children in Hungary: 20 years' experience. *Archives of Gynecology and Obstetrics* 2012; **286**(1): 161-6.

229. Bolen RM, Scannapieco M. Prevalence of child sexual abuse: A corrective metanalysis. *Social Service Review* 1999; **73**(3): 281-313.

230. Devries K, Merrill KG, Knight L, et al. Violence against children in Latin America and the Caribbean: What do available data reveal about prevalence and perpetrators? *Revista panamericana de salud publica = Pan American journal of public health* 2019; **43**: e66.

231. Cénat JM, Amédée LM, Dalexis RD, et al. Sexual Victimization and Sexually Transmitted Infections Among a Nationally Representative Sample of Adolescents and Young Adults in Haiti. *Archives of sexual behavior* 2024.

232. Finkelhor D. The international epidemiology of child sexual abuse. *Child Abuse and Neglect* 1994; **18**(5): 409-17.

233. Gámez-Guadix M, Straus MA, Hershberger SL. Childhood and adolescent victimization and perpetration of sexual coercion by male and female university students. *Deviant Behavior* 2011; **32**(8): 712-42.

234. Hanson-DeFusco J, Dunn WN, Smith EG. Sexual Violence Against Female Children in Liberia: A Cross-Sectional Study of Statutory Rape Prevalence Rates Related to Correlates of Gender Equity. *Journal of Human Rights and Social Work* 2024; **9**(1): 47-70.

235. Levett A. A study of childhood sexual abuse among South African university women students. *South African Journal of Psychology* 1989; **19**(3): 122-9.

236. Magojo TS, Collings SJ. Prevalence and attitudinal predictors of child sexual offending in a nonforensic sample of South African high school males. *Journal of child and adolescent mental health* 2003; **15**(1): 27-32.

237. Singh A, Yiing WW, Nurani NK. Prevalence of childhood sexual abuse among Malaysian paramedical students. *Child Abuse and Neglect* 1996; **20**(6): 487-92.

238. Bossarte RM, Simon TR, Swahn MH. Clustering of adolescent dating violence, peer violence, and suicidal behavior. *Journal of interpersonal violence* 2008; **23**(6): 815-33.

239. da Silva Franzin LC, Olandovski M, Vettorazzi MLT, et al. Child and adolescent abuse and neglect in the city of Curitiba, Brazil. *Child Abuse and Neglect* 2014; **38**(10): 1706-14.

240. DePaul J, Arruabarrena MI, Barbara Torres BT, Ramon Munoz RM. Prevalence of child abuse in Gipuzkoa. *Infancia y Aprendizaje* 1995; **18**(71): 49-58.
